# Mutation adaptive cuckoo search hybridized naked mole rat algorithm for industrial engineering problems

**DOI:** 10.1038/s41598-025-01033-y

**Published:** 2025-06-04

**Authors:** Rohit Salgotra, Supreet Singh, Pooja Verma, Laith Abualigah, Amir H Gandomi

**Affiliations:** 1 Faculty of Physics and Applied Computer Science / Centre of Excellence in Artificial Intelligence, AGH University of Krakow, Kraków, Poland; 2 Faculty of Engineering & Information Technology, University of Technology Sydney, Sydney, Australia; 3 School of Computer Science, UPES, Dehradun, Uttarakhand India; 4 Department of Computer Science & IT, Kathua Campus, University of Jammu, J & K, India; 5 University Research and Innovation Center (EKIK), Óbuda University, Bécsi út 96/B, 1034 Budapest, Hungary; 6 Al al-Bayt University, Computer Science Department, Mafraq, Jordan; 7 Department of Computer Science, Khazar University, Mahsati 41, Baku, Azerbaijan

**Keywords:** Cuckoo search, Naked mole-rat algorithm, Frame design problems, CEC benchmark functions, Mutated cuckoo search, Computational science, Computer science, Engineering

## Abstract

Cuckoo Search (CS) is a popular algorithm used to solve numerous challenging problems. In the present work, a novel variant of CS is presented to eliminate its shortcomings. The proposed algorithm is hybridized with the naked mole rat algorithm (NMRA) to enhance the exploitative behavior of CS, and is called Mutated Adaptive Cuckoo Search Algorithm (MaCN). This new algorithm has self-adaptive properties and its key feature is to divide the solutions into multiple sections, which are often called sub-swarms. In addition, a bare-bones search mechanism is also added to enhance exploration. The use of adaptive inertia weights helps optimize the switching probability, an important CS parameter that helps to achieve a balanced operation. The proposed MaCN algorithm is tested on CEC 2005 and CEC 2014 benchmark problems. Comparative studies showed that MaCN delivers promising results in solving CEC competition benchmark problems compared to JADE, success history-based adaptive DE (SHADE), LSHADE-SPACMA and self-adaptive DE (SaDE), among others. In addition to numerical benchmarks, MaCN is used to solve the industrial engineering frame structure and a comparison with hybridization of particle swarm with passive congregation (PSOPC), shuffled frog leaping algorithm hybrid with invasive weed optimization (SFLAIWO), particle swarm ant colony optimization (PSOACO), early strategy with DE (ES-DE), and others show its superiority. In addition, the Wilcoxon rankum and the Freidmann test statistically prove the significance of the proposed MaCN algorithm. MaCN was found to score first rank for the benchmarks. The application of the MaCN algorithm to solve the design problems of the suggests that the best new results are obtained for all test cases.

## Introduction

There are countless real-world uses of optimization in every area. Many technical and industrial sectors are now struggling to develop a suitable optimizer or algorithm to handle real-world challenges. The primary justification for employing such techniques is that most applications may be characterized as possible domain problems related to the criteria to be achieved. This can be predicted since these methods are used in practically every field of research, including finance, architecture, mathematics, image analysis, weather prediction, industrial engineering, administration, manufacturing, planning, routing issues, pattern recognition, etc. These optimization issues are extremely complicated, making it difficult for academics to solve them quickly. Nature-inspired algorithms (NIAs) have been used in many sectors of research studies in recent years, and now are used in practically every discipline.

NIAs are found to be reliable algorithms for complex optimization problems. Major algorithms include stochastic paint optimizer^1^, sine cosine algorithm (SCA)^2^, flower pollination algorithm (FPA)^3^, differential evolution (DE)^4^, grey wolf optimizer (GWO)^5^, reptile search algorithm (RSA)^6^, harris hawks optimization (HHO)^7^, antlion optimization algorithm (ALO)^8^, naked mole-rat algorithm (NMRA)^9^, gradient-based optimizer^10^, arithmetic optimization algorithm (AOA)^11^, aquila optimizer (AO)^12^, artificial bee colony (ABC) algorithm with local and global information interaction^13^, hybrid SCA^14^, an improved artificial rabbits optimization (ARO) algorithm with chaotic local search and opposition-based learning^15^, seahorse optimization algorithm based on chaotic maps^16^, feature selection using optimization methods^17^, enhanced artificial hummingbird algorithm (AHA)^18^, AHA improved by natural survivor method^19^, dynamic fitness-distance balance-based ARO^20^and adaptive gaining-sharing knowledge algorithm^21^. Although these algorithms have proven extraordinary effectiveness and performance, various assessments show that their performance and competitiveness depend on the tuning parameters used^22,23^. Scaling factor, mutation/crossover rate, switch probability, local/global search, conventional randomization, and population size (*popsize*), are examples of such factors^24^. Early research shows that tuning the parameters of these algorithms requires a trial-and-error search. This procedure takes time and is inefficient. Premature convergence, stagnation of local optima, delayed convergence, and other issues plague these algorithms^22^. Due to these flaws, algorithms are prone to poor solution generation, which results in reduced variety and, as a result, an inability to identify the intended solution^24^. In general, NIAs have been discovered to have flaws and additional effort is needed to develop new algorithms for future scholars.

Cuckoo search (CS) is an effective algorithm introduced in 2009, and has been used in various research domains^25^. Moreover, the basic paper has achieved more than 8,800 citations to date^25^. The algorithm is simple in structure and is considered one of the most promising algorithms among domain researchers because of its linear nature. CS is divided into two parts: exploration (*expl*) via global search and exploitation (*expt*) via local search and a probability of switching ($s_p$) to control *expl* and *expt*operation^26^. The parameter $s_p$ helps the algorithm maintain a proper balance between both *expl* and *expt*. From the literature above and in section 3, it can be seen that most of the work in the literature deals with parametric modifications, and little has been done to improve the *expl* and *expt* simultaneously. There are modifications in the local or global search or a specific switch probability. Apart from that, very little work is available in the literature that improves the algorithm’s overall performance.

Some recent CS adaptations to improve its overall performance aimed at adapting probability, global search, and local search^26^. Another modification was proposed in^27^ that uses adaptive * population* reduction and adaptive switch probability. These modifications make the algorithm adaptive but require new initial and final parameters for adaptations. These parameters make CS complex and highly challenging in execution. Thus, new adaptations are required to the basic algorithms to make them self-adaptive. Apart from that, the hybridization of CS with other algorithms is still a novel concept and has not been explored to its full potential.

Here, a new adaptation of CS is proposed using self-adaptive properties and hybridization concerning naked mole-rat algorithm (NMRA)^9^. The proposed algorithm is named mutation adaptive cuckoo search, inspired by NMRA (MaCN). The proposed MaCN algorithm employs the concepts of iterative division and population division as inspired from^26^. Here, the population division means that the population is split into multiple parts, and each set of these populations are evaluated using different mathematical equations. Iterative division on the other hand deals with multiple set of iterations for finding new solutions. Another modification is adding a bare-bones mechanism for enhanced *expl*, hybridization concerning NMRA for improved *expt*, and inertia weights *iw* associated with switch probability for balancing both *expl* and *expt*operations^28^. The reason for using a bare-bones mechanism is because of its better capabilities to reinforce complementary search for achieving cooperative search^29^. NMRA, on the other hand, is found to provide better *expt*capabilities by enhancing the search space concerning the best solution^9^. Apart from these modifications, adaptive reduction in *popsize*is also followed to minimize the computational burden of the proposed technique^30^.

To check the capability of proposed MaCN algorithm, CEC 2005 dataset^31^and CEC 2014^32^datasets are used. These test functions are highly challenging benchmarks and have been exploited by researchers from various different domains of research. In order to have a fair comparison, more than fifteen recently introduced algorithms namely cuckoo version 1.0 (CV 1.0)^26^, NMRA^9^, memory guided sine cosine algorithm (MGSCA)^33^, beta DE (BDE)^34^, equilibrium optimizer^35^, covariance adaptation based evolution strategy (CMA-ES)^36^, hybridization of SCA with crow search algorithm (SCCS)^37^, adaptive external archive based DE (JADE)^38^, success-history based DE (SHADE)^39^, extended GWO (GWO-E)^40^, self adaptive DE (SaDE)^41^, whale optimization algorithm with opposition based learning (OEWOA)^42^, hybridization of SHADE with CMA-ES (LSHADE-SPACMA)^43^, and fractional-order calculus-based FPA (FA-FPO)^44^ are used.

On the other hand, the algorithms under comparison for CEC 2014 benchmark problems are MGSCA^33^, BDE^34^, population-based incremental learning (PBIL)^45^, chaotic CS (CCS)^46^, improved symbiotic organisms search (ISOS)^47^, random walk GWO (RW-GWO)^48^, blended biogeography-based optimization (B-BBO)^49^, laplacian BBO (LX-BBO)^50^, variable neighbourhood BA (VNBA)^51^, and improved elephant herding optimization (IMEHO)^52^have been used. Apart from that, three industrial engineering problems have also been used to test the performance of MaCN algorithm. Statistical tests (Wilcoxon rank-sum test^53^and Friedman’s test^54^) are done for proving the statistical significance of MaCN. From results in section 5, it can be seen that the proposed MaCN is efficient and is a strong candidate for optimization research. The major highlights of our work are as:

- A hybrid variant of CS using nMRA is proposed to enhance the exploitative properties of CS, named as MaCN.
- New concepts of iterative/generation division is added, by classifing search agents into multiple segments and different mathematical equations for each are used.
- To make the algorithm self-adaptive six mutation operators are applied. It is also taken into account that the added modifications help the algorithm in better exploration and exploitation.
- The size of the population is reduced over the course of iterations, to reduce the computational burden of the proposed algorithm.
- A comparison on CEC 2005, and CEC 2014 is done with respect to JADE, SaDE, SHADE, CMA-ES, LSHADE-SPACMA, LX-BBO, HGAPSO, STMP-SSOA and others, to prove the competitiveness of the proposed algorithm.
- Three industrial engineering frame design problems are also taken into account to prove the significance of the proposed algorithm.

**Fig. 1:**
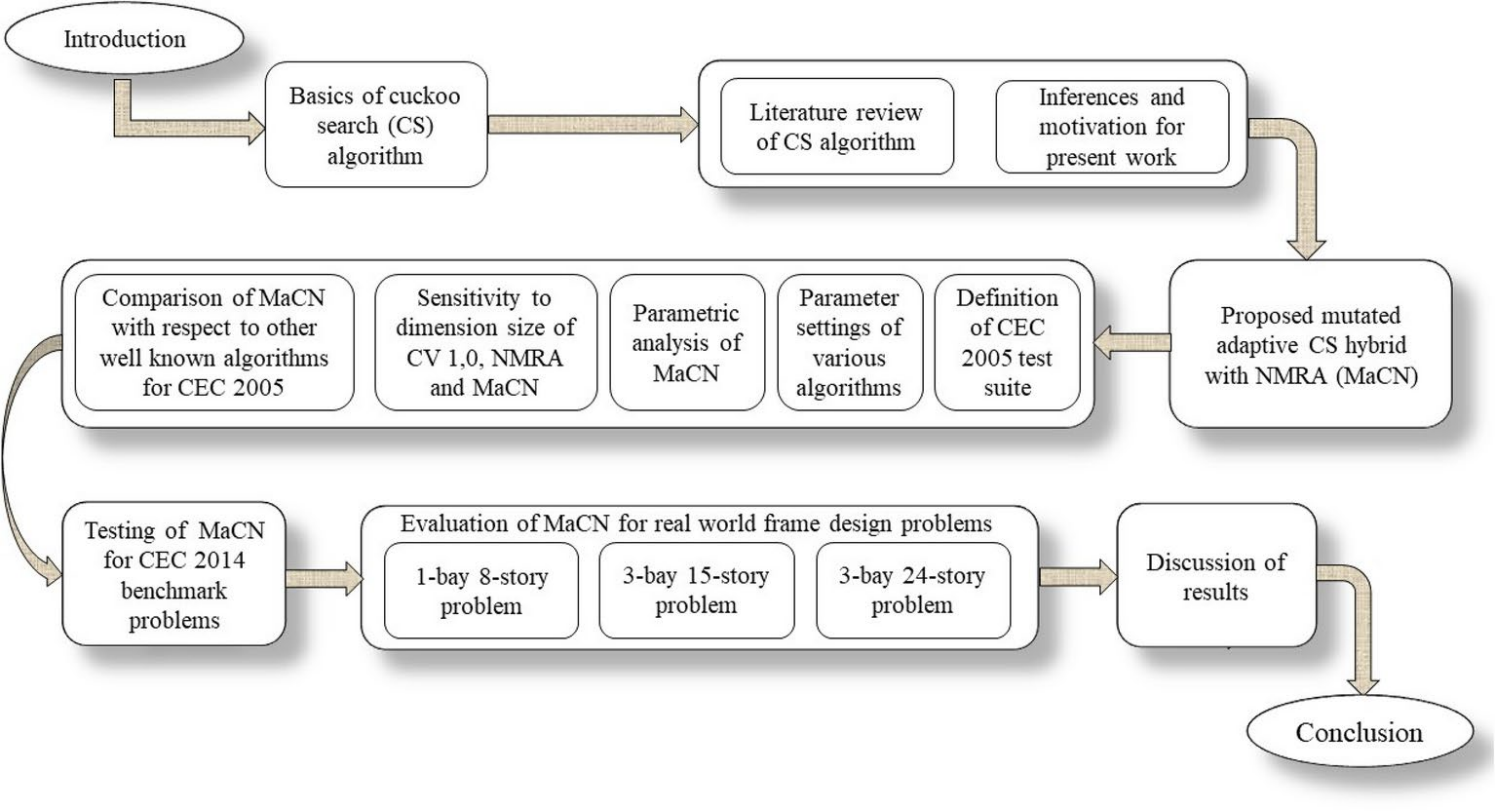
Outline of the article.

Overall, the paper is divided into seven sections. Here the basic concepts of optimization research, the motivation behind present work, and brief details about the present work are presented in section 1. Section 2 gives details about CS, whereas Section 3 has the recent literature on the modifications and application of CS to real applications. Details about the proposal are presented in section 4 and corresponding results are discussed in section 5. The summary of results and insightful implications are discussed in section 6. Finally, the conclusion and future scope are drawn in the final section 7. The outline of the present work is given in Figure 1.

## Basics of cuckoo search algorithm

The cuckoo search algorithm (CS) is a swarm intelligence-based NIA^25^ based on the breeding behaviour of cuckoo species found in nature. The basic idea of CS is discussed as:

### Cuckoos breeding behaviour

Cuckoos are prevalent birds due to the beautiful voice and strategy of reproduction they are used. The cuckoo species like *ani* and *guira*follow brood parasitic behaviour by placing their eggs in other species nests. Although cuckoo species enhance the probability of hatching of their eggs by removing the eggs of another species^55^. If the other species or host birds found that eggs placed in the nest are not their own, then host birds either throw the eggs away or dump the whole nest. Some species of cuckoo are experts in mimicry of pattern and colour of some host eggs. By this, the abandoned rate of the cuckoo eggs is reduced, and the reproduction rate increases. Further, parasitic cuckoos usually select a nest in which the host species just lay down eggs. This is because the cuckoo eggs hatch comparatively faster than the host eggs and after hatching, it throws out host eggs. So, the cuckoo chick gets more food from the host^55^. Sometimes, the cuckoo chick sometimes mimics the host chick’s voice to enhance the chance of getting more food.

### Lévy flights

In nature, most of the birds follow random walks to find food. In the same way, cuckoos also follow random walks where the following location depends on the transition probability and current location. The different studies are given in^56^^57^shows that the behaviour of main birds illustrates the Lévy flight characteristics. The use of Lévy flights enhances the capability of searching in comparison with a simple random walk. It is also a random walk where length of steps follows a heavily-tailed distribution function^25^. Due to this behaviour, it has been used for optimization, and early-stage results prove its capability for optimal search^58^.

### Cuckoo Search

Three ideal basic rules are utilized to define the CS algorithm^25^, such as:

- One cuckoo only lays one egg, which they find at random in a nest.
- The best eggs are chosen and passed down to future generations.
- The value of host nests is constant, and the host probability $p \in [0,1]$ to recognize cuckoo eggs. The host bird either discard the cuckoo egg, or quit the entire nest and build a new nest. It’s also believed that some *n* nests are replaced with new nests, for diversity.

Cuckoo search can be implemented using the three rules stated above, with each cuckoo representing a possible solution. Using the Lévy flight, a new nest or solution $x_i^{t+1}$ for the $i^{th}$ cuckoo is generated.

1 $$\begin{aligned} x_i^{t+1}=x_i^{t}+\alpha \hspace{2pt}\otimes Levy (\beta ) \end{aligned}$$

where, $x_i^{t}$ is a current solution to find the next solution $x_i^{t+1}$, $\alpha>0$ is the step size corresponds to the scale of problem under consideration and $\otimes$ defines element wise multiplication. The main objective to find a new solution (cuckoo) and possibly a better solution to replace not good solution. Here, Lévy flight based random walk is preferred because it explores effectively. The Lévy flight step size is much longer and derived from the Lévy distribution having an infinite mean and variance.

2 $$\begin{aligned} Levy\sim \mu =t^{-\beta },(1<\beta \le 3) \end{aligned}$$

where $\beta$ is the probability of an event occurring. Some new solutions acquired by Lévy flights are close to the best solution ever developed, and they improve the speed of local search. However, far-field optimization should be used to define a portion of new solutions, and the position of these solutions should be away from the present best to prevent the system from becoming stuck in local minima. The steps of cuckoo search are based on a random walk inspired by power-law distribution, resulting in a heavy-tailed step size. Randomization on a larger scale allows for such a large step size. The literature review is detailed in the next section.

## Literature review

In this section, a detailed literature on CS and inference drawn from the literature has been discussed. The detailed literature is broadly categorized into two parts: a literature based on modifications of CS and a literature based on applications of CS.

### Literature based on modifications

Cuckoo search is a very promising algorithm for different fields of optimization. In^59^a binary CS with sigmoid function was proposed to solve binary optimization problems. In addition, discrete versions of CS (DCS) have been proposed by^60,61^to tackle the traveling salesman problem (TSP). The DCS algorithm proposed by^61^uses population rebuilding to improve its efficiency compared to discrete PSO^62^. The modified CS algorithm (MCS) in^63^was used to solve the unconstrained optimization problems. As the author tested the performance of MCS by applying only on eight benchmark functions, it can be analyzed that functions providing good results are considered, and the rest are neglected. The other version of MCS was implemented^64^ with rough sets to handle high dimension data for feature selection. This version is meant to reduce learning parameters so that consumption time decreases and convergence speed increases.

In addition, Abdul et al.^65^proposed another version of MCS to suppress the side lobes in the linear antenna array. The other variant of MCS was implemented in^66^ to solve analytical aspects of multiobjective distribution systems with different compensators. Here, the basic CS algorithm was modified using a crossover operator to maintain a balance between *expl* and *expt*. This leads to an improvement in the convergence rate and quality of the solution. The one-rank CS algorithm^67^ was proposed for the optimization of the algorithmic trading system. In this, a new solution was generated by combining the phases *expl* and *expt* and the best utilization of Lévy flight such as step size $\alpha =0.01$. The performance of basic CS was enhanced by a new variant of MCS^68^to optimize the wired-EDM process. CS was modified^69^to converge faster, although it is not guaranteed that the rate of convergence is fast because it is based on random walks. The other variants of CS consist of improved CS (ICS) by modify step length for solving coloring problem of planar graph^70^, new CS with improved *expl* and *expt*properties for numerical optimization^26^, Chaotic CS to reconstruct chaotic dynamics^71^, enhanced CS for contrast management of grey scale images^72^, Complex valued CS to optimize design problems^73^, novel CS-based on Gauss distribution (GCS)^74^, quantum CS for clustering data problems^75^, new CS integrated with fuzzy system^76^, hybrid multi-objective CS based on dynamical local search^77^, improved CS integrated with chaotic flower pollination algorithm to maximize coverage area of wireless sensor network^78^, hybrid CS with GWO for medical image fusion^79^, hybridization of CS with BA for optimization of numerical benchmark functions^80^, hybrid CS with krill herd optimization algorithm for engineering problems^81^, fractional-order CS to identify parameters of financial system^44^, hybrid CS with Levenberg-Marquardt back propagation algorithm^82^and it can be seen that local optima stagnation problem will be avoided. Hybrid CS with genetic algorithm (CS-GA) was proposed to solve redundancy allocation and reliability problems^83^, drilling path optimization problem for PCB holes^84^and hole making sequence optimization problem^85^. CS was also hybridized with differential evolution (CS-DE) for planning optimization problem of uninhabited combat air vehicle (UCAV)^86^, solving constrained engineering problems^87^and three-dimensional path planning problem of UCAV^88^. Hybrid CS with ant colony optimization (CS-ACO) to solve routing problem of mobile ad hoc network^89^, and job scheduling problem^90^. The population-based Harmony search algorithm is hybridized with CS (CS-HS) for the optimal water distribution system design^91^, and continuous optimization problems^92^. The CS was also hybridized with other local search-based algorithms such as CS with tabu search algorithm to solve quadratic assignment problems^93^, CS with quantum computing for knapsack problems^94^, CS with Nelder-Mead method for performance optimization of multi-cell solar system^95^, CS with newton method to solve linear least square problem^96^and CS with GWO to extract parameters of photo voltaic models^97^.

### Applications of CS algorithm

Apart from the modifications, CS is found to be capable for solving real optimization problems. These problems include diagnosis of diabetes^98^, clustering of web documents^99^, image recognition^100^, low contrast satellite images quality improvement^101^, structure design problems^102^, embedded system design problem^103^, designing of wind power system^103^, optimization of surface roughness in laser cutting^104^, power loss reduction of distribution system^105^, design of computer-aided power system^106^, economic load dispatch problem^107^, twitter sentiment analysis^108^, movie recommender system^109^, higher order filter design^110^and multi-machine power system stabilizer design problem^111^. The other applications of CS are forecasting of electrical load for power supply system^112^, optimization of visible light communication coverage in smart homes^113^, multi-objective hydro-thermal scheduling^114^, thinning of circular antenna arrays^115^, optimize efficiency of home energy management system^116^, detection of vibration fault for hydro-electric generating unit^117^, multi-objective community detection for dynamic networks^118^, solving non-linear equations of systems^119^, optimized configuration of electric distribution network^120^, waste management in smart cities^121^, navigation of mobile robots in uncertain environment^122^, gathering of load-balanced data^123^, prediction of defect in software data sets^124^, web service composition^125^, designing of hydraulic damper^126^and optimization of water distribution system^127^.

### Motivation for present work

Though, a lot of articles have been published on basic CS^128^. However, most of the work on the application part presented only minute details about the algorithms, and very limited research work has been done on parametric enhancements^129^. Thus posing a serious challenge on the proper *expl* and *expt*properties of CS algorithm. Apart from that, most of the hybridization’s and improved version of CS deals with the change in one or two parameters and not necessarily the whole algorithm^26^. The major drawback of these algorithms is that, most of the proposed improvements are based on the basic CS and not on the enhanced versions. Thus there is a need to propose new hybrid versions of CS algorithm. Also recent studies on iterative division and population adaptation has brought significant advancements in the performance of CS algorithm^40^. All of the above said issues have motivated the authors to propose a new improvement in CS and its overall performance. In the next section, why’s and how’s of the proposed MaCN are discussed in details.

## The proposed MaCN algorithm

CS is one of many researchers’ most promising optimization algorithms. Numerous works have been done to enhance its performance and solve real complex optimization problems. This algorithm is more suitable when dealing with higher dimension problems of optimization with additional improvements. As per no free lunch theorem^130^, no algorithm is considered a generic problem solver, so it motivates the researchers to hybridize and introduce modifications in CS. These modifications enhance the performance of an algorithm and make it capable of solving computationally complex problems. The classical CS suffers from the problems of stagnation and poor switching between *expl* and *expt*phases^129^. In a general CS, four parameters are of significant concern and need to be enhanced for better algorithm performance. Among these four parameters, scaling factor, switch probability (*pa*), $\epsilon$ parameter of local random walk is the third parameter, and *popsize* of search agents is the final parameter. As discussed in section 3.3, all these four parameters have not been exploited to their full potential, and there is still scope for modification.

In the present study, the above discussed four parameters are adaptive instead of static nature, and based on this new algorithm has been proposed. The main aim of this newly proposed algorithm to mitigate the problems of poor *expt* and *expl*, slow convergence speed, and improper balancing between local search & global search. This algorithm is considered an extended version of CV 1.0 and named mutation adaptive CS hybrid with NMRA (MaCN). This algorithm is also based on the iterative/generation division concept^26^. In the division of generations concept, the population of search agents is classified into multiple segments and different mathematical notations define each segment. Here, the proposed algorithm follows the dual iterative division and broadly splits into global search phase (*gsp*) and local search phase (*lsp*). The different phases of the proposed algorithm and various modifications are discussed in consecutive subsections.

### Initialization

The first phase of MaCN is to deals with initialization of *N* cuckoos (population) in the random manner for dimension *d* of a problem. This phase is mathematically modelled as:

3 $$\begin{aligned} x_{i,k}=x_{min,k}+r(0,1) \times (x_{min,k}-x_{max,k}) \end{aligned}$$

where, *i* corresponds to range [1, 2,.....*n*], *k* defines for [1, 2,...*d*], $x_{i,k}$ represents $i^{th}$ solution for the $k^{th}$ dimension; *r*(0, 1) is a random number between [0,1]; $x_{min,k}$ and $x_{max,k}$ denotes lower and upper boundary conditions. This phase is kept same during the execution of complete algorithm and incorporate major changes from the first generation.

### Global search phase

In the *gsp* of the proposed algorithm, for first half of the generations, original equation of basic CS are used. The equation used to represent the *gsp* of basic CS is defined as:

4 $$\begin{aligned} x_{i}^{t+1}=x_i^t+\alpha \otimes {Levy(\beta )}(x_{best}-x_i^t) \end{aligned}$$

where $x_{i}^{t+1}$ is the new solution for current iteration, $x_i^t$ defines the solution generated in previous iteration, $\otimes$ symbol denotes the entry wise multiplication, $x_{best}$ is current best and step size $\alpha> 0$ is defined by dimension *d* of the problem. The randomization used here is based on Lévy distribution and generated by equation (2).

Further, a new modification based on the bare-bones variant has been added in equation (4) of classical CS. The main aim of using this mechanism to generate new, highly diverse solutions and helps in improving the performance of algorithms during *expl* operation. The modified mathematical equation of the *gsp* then becomes,

5 $$\begin{aligned} Gx_{i,d}= W_r \times x_{new}^{t+1}+(1-W_r)\times Cx_{i,d} \end{aligned}$$

for $$Cx_{i,d}=n(\frac{x_{p,d}^t+q_{k,d}^t}{2},|s_{p,d}^t-x_{q,d}^t|)$$

where $Gx_{i,d}$ denotes the modified solution in *gsp* for *d* dimension of the problem, $W_r$ is coefficient of weight defined in stochastic manner with range [0,1], $x_{p,d}^t$ & $x_{q,d}^t$ are two randomly selected solutions from entire population for problem dimension *d*and all the remaining notations used here have same relevance as provided in basic CS. The bare-bones mechanism is commonly referred to as cooperative search mechanism and used in PSO^29^. This mechanism uses the collaborative benefits of different search operators and hence controls the problems that occur due to one search operator. The bare-bones parameter can generate large step size during the initial iterations so that the algorithm performs better for *expl* operation. Thus, this mechanism is introduced to provide large step sizes and enhance the *expl* process of MaCN.

For the second set of iterations, more heed is paid to the *expt* operation of the algorithm and formulate new equations to make it capable for finding optimal solution. Here, the new equations have been derived from original *gsp*equation of CS in combination with GWO algorithm^5^. These equations are used to generate new solution and mathematically defined as:

6 $$\begin{aligned} g_1=x_i-M_1(N_1.x_{new}-x_i^t);\hspace{5pt} g_2=x_i-M_2(N_2.x_{new}-x_i^t);\hspace{5pt} g_3=x_i-M_3(N_3.x_{new}-x_i^t) \end{aligned}$$

7 $$\begin{aligned} x_{new}^{t+1}=\frac{g_1+g_2+g_3}{3} \end{aligned}$$

Here the new solution is denoted by $x_{new}$ for current iteration value and $M_1$, $M_2$, $M_3$ and $N_1$, $N_2$, $N_3$ are derived from *M* and *N* respectively. The definition of *M* and *N* are given by:

8 $$\begin{aligned} M=2m.r_1-m; \hspace{5pt} N=2.r_2 \end{aligned}$$

where *m* decreases linearly from 2 to 0 by changes its value in accordance with iterations, $r_1$ & $r_2$ randomly distributed in range [0,1]. This new equation is utilized due to better *expt* properties of GWO so that the algorithm is able to exploit potential solutions in *gsp*.

### Local search phase

The *lsp* of original CS mainly deals with local random walk of search agents and governed by equation (9)

9 $$\begin{aligned} x_{i}^{t+1}=x_i^t+\alpha \otimes (\epsilon )\otimes (x_p^t-x_q^t) \end{aligned}$$

where $x_p^t$ and $x_q^t$ are random solutions from the whole population for $t^{th}$ iteration and the rest of the notations are the same as the *gsp*. The $\epsilon$ parameter is randomly distributed between 0 and 1.

In the present work, only $\epsilon$ parameter of equation (9) has been replaced by scaling factor (*S*)^131^ for first half of the iterations and new parametric equation is defined as:

10 $$\begin{aligned} S_i^{t+1}= {\left\{ \begin{array}{ll} \frac{1}{2}\times (sin(2\pi \times f_{req} \times t + \pi )\times \frac{t_{max-t}}{t_{max}} +1);\hspace{10pt} if \hspace{5pt} r> 0.5 \\ \frac{1}{2}\times (sin(2\pi \times f_{req} \times t)\times \frac{t_{max-t}}{t_{max}} +1); \hspace{25pt} if \hspace{5pt} r < 0.5\\ \end{array}\right. } \end{aligned}$$

where, $f_{req}$ is the frequency of sine signal, *t* and $t_{max}$ is the current and maximum iterations.

For the second iterative half, the MaCN algorithm is governed by a new equation. This new equation is used from breeder phase of NMRA^9^. From the original paper of NMRA, it has been found that NMRA exhibits good *expt* properties, and due to these properties, the breeder phase equation of NMRA is added in this phase for the second iterative half. The mathematical description of this equation is given as:

11 $$\begin{aligned} x_{i}^{t+1}=(1-\lambda )x_i^t+\lambda (x_{best}-x_i^t) \end{aligned}$$

$\lambda$ is responsible for controlling the frequency of mating with queen and based on simulated annealing (*sa*) *iw*. A detailed description of *sa* *iw* is given in the parameter adaptation subsection. All the other notations used in equation (11) have the same meaning as used in the *gsp*.

### Population adaptation

The *popsize* is a crucial parameter for optimization. When solving optimization problems, *popsize* multiplied by maximum iterations, gives the total function evaluations. It is important to remember that the bigger the *popsize*, the larger is the total number of function evaluations, and that a smaller *popsize*, while having fewer function evaluations, may result in local optima stagnation. A diminishing population strategy based on^30^ is used. This method aids in initial *expl* with a full *popsize*, and a limited population method leads to better solutions in the final stages. It also aids in the provision of better convergence properties and the discovery of new global optimum solutions without sacrificing the previous global best. The general equation for population adaptation is given by:

12 $$\begin{aligned} N_{t+1}= {\left\{ \begin{array}{ll} (1-\Delta f_t^{best})N_t, \hspace{10pt} if \Delta f_t^{best}\le \Delta f_{max}^{best}\\ (1-\Delta f_{max}^{best})N_t, \hspace{10pt} if \Delta f_t^{best}> \Delta f_{max}^{best}\\ {min}_{N}, \hspace{10pt} if N_{t+1} <{min}_{N} \end{array}\right. } \end{aligned}$$

where $N_{t+1}$ is the *popsize* at generation *t*, $\Delta f_t^{best}$ is given by $(\frac{f_{t-1}^{best}-f_{t-2}^{best}}{|f_{t-2}^{best}|})$ is the change in the best fitness, $\Delta f_{max}^{best}$ is the threshold. Also, a minimum *popsize* has been established to mitigate the negative consequences of smaller population levels. The overall shape is an exponential curve at first, followed by a steady state, and finally an exponential drop. It’s worth noting that there’s no need to define a user-based pattern.

### Parametric adaptations

Overall, in present work there are two major parameters; i) Switch probability (*pa*) of CS algorithm, ii) NMRA’s mating factor $(\lambda )$. These parameters are made adaptive with the help of five *iw*. These *iw* help in balancing *expl* and *expt*phases of algorithm^132^. Here, five types of *iw* namely simulated annealing (*sa*) *iw*, exponential decreasing (*exp*) *iw*, linear decreasing (*linear*) *iw*, chaotic *iw* and logarithmic decreasing (*log*) *iw* are used for parametric analysis of MaCN algorithm. The mathematical description of these *iw* are presented in Table 1

**Table 1:** Description of various inertia weights.

S.No	Inertia Weight	Mathematical Description	Initial values	Significance
1	*sa*^133^	$\gamma _k= \gamma _{min}+(\gamma _{max}-\gamma _{min})\times r^{(k-1)}$	$\gamma _{max}$, $\gamma _{min}$, *k* = *rand*[0, 1]	Improve convergence
			$r=0.95$	rate
2	*exp*^134^	$\gamma (iter)=\gamma _{min}+(\gamma _{max}-\gamma _{min})exp\left[ -\frac{iter}{(\frac{t_{max}}{10})}\right]$	$\gamma _{max}$, $\gamma _{min}$ = *rand*[0, 1]	Algorithm convergence
			*iter*= current iteration	faster during initial
			$t_{max}$= maximum iterations	stages
3	*linear*^135^	$\gamma _k= \gamma _{max}-\left( \frac{\gamma _{max}-\gamma _{min}}{t_{max}} \right) \times k$	$\gamma _{max}$, $\gamma _{min}$, *k* = *rand*[0, 1]	Algorithm work efficiently
			$t_{max}$= maximum iterations	in *expt* phase
4	*chaotic*^136^	$\gamma _k=(\gamma _1-\gamma _2)\times \frac{t_{max}-iter}{t_{max}}+\gamma _2 \times k$	$\gamma _1=0.9$; $\gamma _2=0.4$	Explore search space
			$k=4 \times k \times (1-k)$	efficiently
5	*log*^137^	$\gamma (iter)= \gamma _{max}+(\gamma _{min}-\gamma _{max})\times log_{10} \left( k+\frac{10\times iter}{t_{max}} \right)$	$\gamma _{max}$, $\gamma _{min}$, *k* = *rand*[0, 1]	Avoid local optima
			*iter*= current iteration	stagnation problem
			$t_{max}$= maximum iterations	

### Greedy selection

**Fig. 2:**
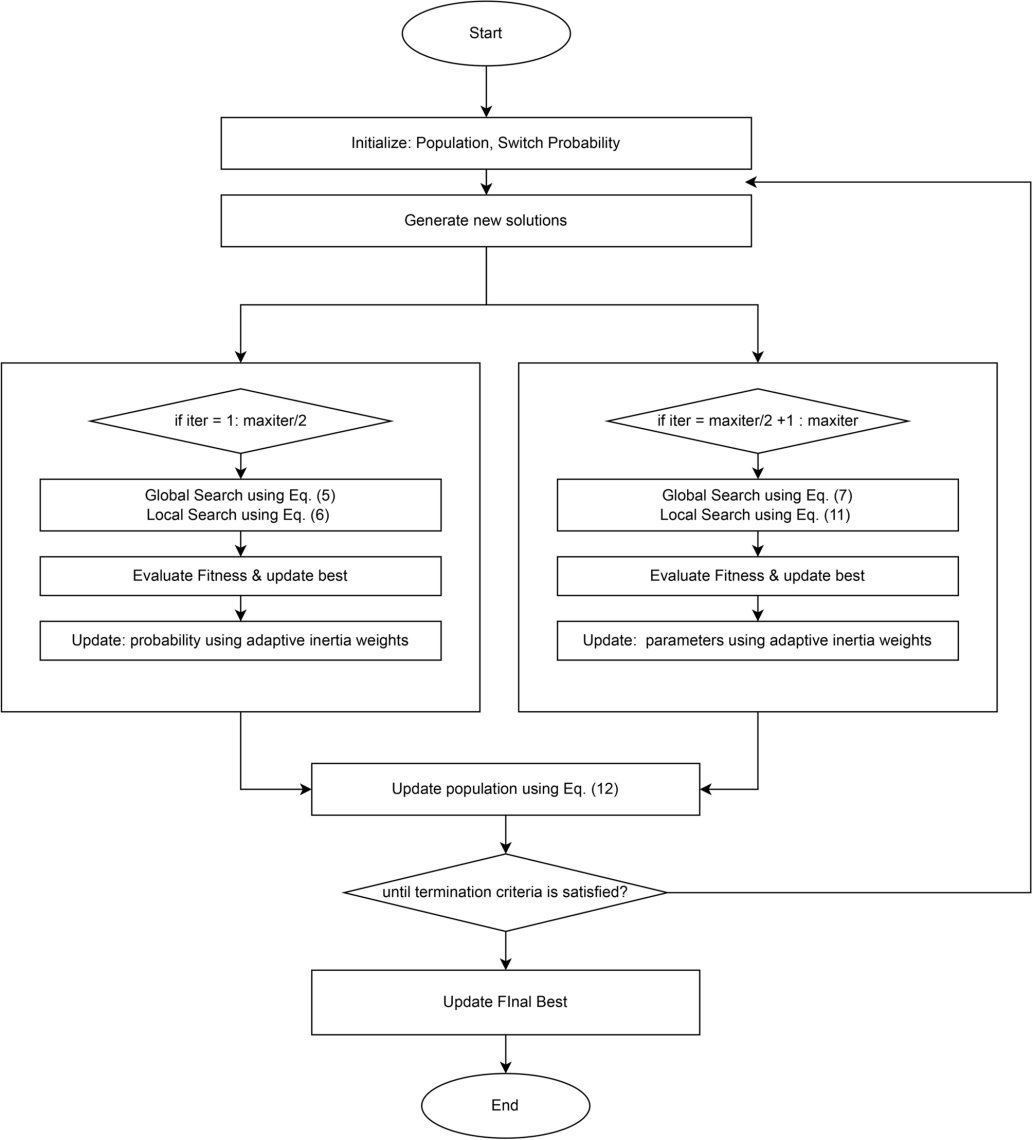
Flow Chart of the proposed MaCN algorithm.

The final phase of optimization algorithm is selection of newly generated solution. For proposed algorithm MaCN, selection procedure is greedy in nature and newly generated solution is compared with solution of previous generation. So, if the new solution is more fit than the previous solution, then the new solution is replaced by the previous solution. The mathematical equation to present greedy selection procedure is given as:

13 $$\begin{aligned} S_{new}^{t+1}= {\left\{ \begin{array}{ll} S_{new} \hspace{20pt}if f(S_{new})<f(S_i^t)\\ S_i^t \hspace{35pt} otherwise \end{array}\right. } \end{aligned}$$

where $S_{new}^{t+1}$ is the solution generated in current $t+1$ iteration, $S_i^t$ defines the solution generated in previous iteration and $f(S_i^t)$ represents the fitness value for $S_i^t$ solution. The pseudocode of the proposed MaCN is presented in Algorithm 1, and the flow chart is given in Figure 2.

**Algorithm 1:**
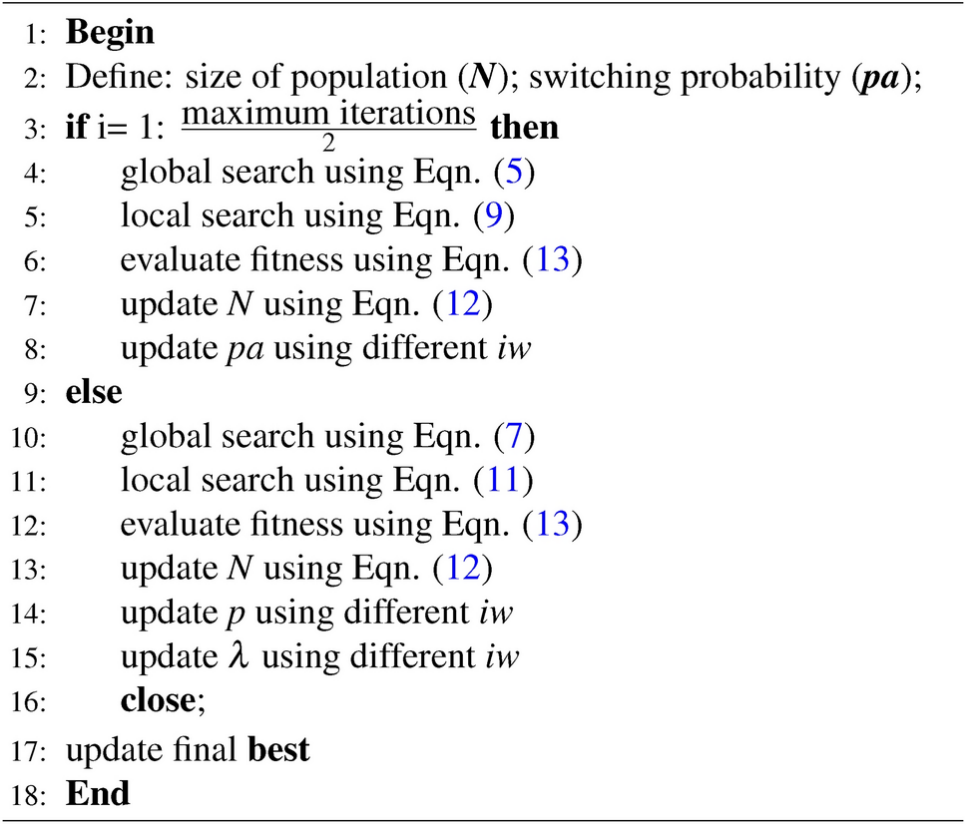
Pseudocode of proposed MaCN algorithm

### Computational complexity

The complexity of the developed MaCN is based on the complexity of traditional CS, NMRA, and bare-bones, and it is given as follows.

14 $$\begin{aligned} O(MaCN)=(N)\times O(CS)\times (O(NMRA)+O(bare-bones)) \end{aligned}$$

where, the time complexity of the used CS optimizer is given in Equation (15), for NMRA is shown in Equation (16), and for bare-bones is given in Equation (17).

15 $$\begin{aligned} O(CS)=O(N\times (t\times Dim+1)) \end{aligned}$$

16 $$\begin{aligned} O(NMRA)=O(N\times Dim) \end{aligned}$$

17 $$\begin{aligned} O(bare-bones)=O(N\times Dim) \end{aligned}$$

Therefore, the complexity of the MaCN is given.

18 $$\begin{aligned} O(MaCN)=O\left( t\times N \times \left( Dim+N\right) \right) \end{aligned}$$

where, *t* is the iteration counter, *N* as the maximum solutions, and *Dim* presents the problem dimension.

It is clear from the time complexity analysis that the proposed MaCN does not take more complexity compared to other basic methods. The primary process, like the solutions initialization and solutions evaluation, is almost the same as the basic method. However, the maximum time complexity of MaCN is acceptable and does not exceed other methods.

## Results and discussion

Here, to analyse the performance, two datasets (CEC 2005 and CEC 2014) from CEC competitions and steel frame design optimization problems are taken into consideration. The section is broadly classified into 7 subsections. The definition of CEC 2005 test suite is presented in the first subsection, and detail regarding various parameters of different NIAs is given in the second. The parametric analysis of the proposed MaCN is in the third subsection. In the fourth subsection, sensitivity to dimension size has been analyzed for 5 different values. After performing the parametric and dimensional analysis, the fifth and sixth subsections deal with the performance analysis of the proposed MaCN for the CEC 2005 test suite and for the highly complex CEC 2014 test suite. The seventh subsection deals with a comparative analysis of real frame design optimization problems.

All the results are simulated on HP 14 notebook, AMD Ryzen 5 processor, 8GB RAM, 64-bit Windows 10 and MATLAB 2016a.

### Description of CEC 2005 test suite

This section gives the definition of CEC 2005 test suite^31^ to test the performance of MaCN. This test suite mainly consist of two types of functions as presented in Table 2. Firstly, Unimodal functions (*G*1 to *G*7) having single global minimum, are used to check algorithm effectiveness to perform the *expt* phase. In order to check the convergence of the algorithm for *expl* operations, multi-modal benchmarks (*G*8 to *G*12) are used. The optimum fitness value of all test functions is listed in the last column of Table 2 and taken as $f_{min}=0$.

**Table 2:** Description of CEC 2005 test functions.

Function	Dim	Range	Shift position	$f_{min}$
Uni-modal functions				
G1(q)= $\sum _{i=1}^{n} q_i^2$	30	$[-100,100]$	$[-30,-30,..,-30]$	0
G2(q)= $\sum _{i=1}^{n} |q_i|+\Pi _{i=1}^n |q_i|$	30	$[-10,10]$	$[-3,-3,..,-3]$	0
G3(q)= $\sum _{i=1}^{n}(\sum _{j-1}^i q_j)^2$	30	$[-100,100]$	$[-3,-3,..,-3]$	0
G4(q)= $max_i\{|q_i|, 1\le i \le n\}$	30	$[-100,100]$	$[-3,-3,..,-3]$	0
G5(q)= $\sum _{i=1}^{n-1} 100(q_{i+1}-q_i^2)^2+(q_1-1)^2$	30	$[-30,30]$	$[-3,-3,..,-3]$	0
G6(q)= $\sum _{i=1}^{n}([q_i+0.5])^2$	30	$[-10,10]$	$[-3,-3,..,-3]$	0
G7(q)= $\sum _{i=1}^{n} iq_i^4 + random [0,1]$	30	$[-1.28,1.28]$	$[-3,-3,..,-3]$	0
Multi-modal functions				
G8(q)= $\sum _{i=1}^{n} [q_i^2-10cos(2\pi q_i)+10]$	30	$[-5.12,5.12]$	$[-30,-30,..,-30]$	0
G9(q)= $-20exp(-0.2 \sqrt{\frac{1}{n}}\sum _{i=1}^n q_i^2)-exp(\frac{1}{n}\sum _{i=1}^n cos(2\pi q_i))+20+e$	30	$[-100,100]$	$[-30,-30,..,-30]$	0
G10(q)= $\frac{1}{4000}\sum _{i=1}^N q_i^2 - \Pi _{i=1}^N cos(\frac{q_i}{\sqrt{i}})+1$	30	$[-600,600]$	$[-30,-30,..,-30]$	0
G11(q)= $\frac{\pi }{n}10sin(\pi p_1)+\sum _{i=1}^n-1(p_i-1)^2[1+10sin^2(\pi p_{i+1})]$	30	$[-50,50]$	$[-30,-30,..,-30]$	0
$${(p_n-1)^2+\sum _{i=1}^u(q_i,10,100,4) p_i=1=\frac{q_i+1}{4}}$$				
G12(q)= $0.1 ( \sin ^2(3\pi q_1)+\sum _{i=1}^n(q_i-1)^2(1+\sin ^2(3\pi q_i+1)))$	30	$[-50,50]$	$[-30,-30,..,-30]$	0
$$+0.1((q_n-1)^2[1+\sin ^2(2\pi q_n])+\sum _{i=1}^n u(q_i, 5, 100, 4)$$				

### Parameter settings

To test the efficiency of proposed MaCN for two test suites (CEC 2005 and CEC 2014), its statistical results are analysed with other improved algorithms. In case of CEC 2005 test functions, algorithms used for comparison are CV 1.0^26^, NMRA^9^, MGSCA^33^, BDE^34^, EO^35^, SHADE^39^, CMA-ES^36^, LSHADE-SPACMA^43^, SCCS^37^, GWO-E^40^, SaDE^41^, JADE^38^, OEWOA^42^and FA-FPO^44^. On the other hand, the algorithms under comparison for CEC 2014 benchmark problems are MGSCA^33^, BDE^34^, CCS^46^, VNBA^51^, ISOS^47^, RW-GWO^48^, B-BBO^49^, LX-BBO^50^, PBIL^45^and IMEHO^52^.

The various parameters of all the above mentioned algorithms are selected from their recent literature and presented in Table 3. In the case of CV 1.0, the important parameter is switch probability $(pa) = 0.5$. Apart from this, one more parameter $\vec {\alpha }$ of GWO is added in CV 1.0 to control the *expl* and *expt* phases, which is linearly decreasing from 2 to 0. For NMRA, breeding probability (*bp*) and mating factor $(\lambda )$are two important parameters and value of these parameters are same as^9^. A detailed list of all the parameters is given in Table 3.

**Table 3:** Parameter selection of various algorithms under comparison.

Algorithm name	Parameters involved
CV 1.0^26^	$\vec {\alpha }$=Linearly decreasing [2,0]; Switch Probability (*pa*)=0.5
OEWOA^42^	$\vec {\alpha }$ = Exponentially decreasing function; $b=1$
SCCS^37^	$r_1, r_2, r_3 = [0,1]$
PBIL^52^	Learning rate (*LR*)=0.1; Mutation probability $(p_m)$=0.02
ISOS^47^	$q \in [1,100]\%$, $r \in [0,1]$
B-BBO^50^	$H=1$; $I=1$
GWO-E^40^	$\vec {\alpha }$ = Linearly decreasing from 2 to 0
FA-FPO^138^	$\alpha = [0.1,1]$, $S=adaptive$
IMEHO^52^	w= Linearly decreasing in range [0.9,0.2]; $$\alpha$$$$\epsilon$$ [0,1]; Probability $$(p_c)$$=0.05
SaDE^41^	$F, CR=$ self adaptive
SHADE^35^	$P_{best}=0.1$, *ARC* *rate*= 2
LX-BBO^50^	$H=1$; $I=1$
LSHADE-SPACMA^35^	*c*=0.8, $P_{best}=0.11$, *ARC* *rate*= 1.4, *FCP*=0.5
JADE^38^	$F=0.5$; $CR=0.9$; $1/c=[5,20]$; $p=[0.05,0.20]$
EO^35^	$a_1=2$, $a_2=1$, $sign(r-0.5)$
VNBA^52^	Loudness (*A*)=0.5; Pulse rate (*r*)=0.5
RW-GWO^48^	$\vec {\alpha }$ = Linearly decreased from 2 to 0
CCS^52^	Switch probability (*pa*)=0.25
NMRA^9^	$bp=0.5$; $\lambda =[0,1]$;
BDE^34^	*F*, *CR* = Beta distribution
MGSCA^33^	$r_1$ = adaptive; $r_2$ = [0,2$\pi$]; $r_3$ = uniformly distributed [0,2]
MaCN	Switch probability (*pa*)= Exponential decreasing; mating factor ($\lambda$)=sa; Population=*adaptive*

### Parametric analysis of MaCN

The parametric analysis of MaCN has been provided in this subsection. The MaCN algorithm has basically two parameters of major concern, namely the switch probability (*pa*) of the CS algorithm and the mating factor $(\lambda )$ of NMRA. To perform analysis on these parameters, three different constant values (0.25, 0.50 & 0.75) and five different adaptive *iw* (*sa*, *exp*, *linear*, *chaotic* & *log*) are used for 7 uni-modal benchmarks (*G*1 to *G*7) and 5 multi-modal benchmarks (*G*8 to *G*12) of CEC 2005^31^ test suite. The results are simulated for 30 dimension size, 500 iterations and presented in mean & standard deviation (*std*) of 51 runs as in Table 4. So the total number of function evaluations in this case are $30 \times 500 = 15000$. The dependence of these parameters on the performance of MaCN are discussed as:

*Analysis of CS parameter:* The important (*pa*) parameter of CS algorithm controls *expl* and *expt* operations. Here, three different constant values and five different as+daptive *iw* are associated with this parameter for analyzing the performance of MaCN. From the Table 4, it has been analyzed that parameter *pa* with *exp*, and *log* *iw* provide almost same and best results for all the benchmark functions. So, finally *exp* *iw*-based *pa* parameter has been selected for further simulations after performing detailed analysis for MaCN algorithm.

*Analysis of NMRA parameter:* The main parameter of NMRA is $(\lambda )$ and found useful in the implementation of *lsp* equation of the proposed MaCN algorithm. Here, ability of proposed MaCN algorithm has been analyzed for three constant values and five *iw* applied to this parameter and is given in Table 4. Here we find that results are similar for all the cases and efficiency of MaCN algorithm is not much affected by variation in $\lambda$ parameter. However, finally, *sa* *iw* is considered for this parameter because *sa* *iw* helps the algorithm explore a particular area of search spacein exploring certain sections.

**Table 4:** Parametric analysis of MaCN.

Simulation results for different cases of CS switching probability parameter (*pa*)									
Function		$pa_{0.25}$	$pa_{0.50}$	$pa_{0.75}$	$pa_{sa}$	$pa_{exp}$	$pa_{linear}$	$pa_{chaotic}$	$pa_{log}$
G1	Mean	3.49E-269	3.63E-262	5.37E-256	1.37E-251	5.91E-264	4.51E-256	3.46E-256	1.25E-263
	Std	0	0	0	0	0	0	0	0
G2	Mean	1.20E-134	1.41E-131	1.29E-129	2.05E-126	9.76E-132	1.33E-128	1.02E-128	4.80E-132
	Std	3.77E-134	9.25E-131	4.85E-129	1.00E-125	6.22E-131	8.36E-128	4.25E-128	0
G3	Mean	6.33E-266	5.04E-263	3.11E-257	3.82E-251	1.07E-262	2.12E-255	1.95E-255	3.93E-264
	Std	0	0	0	0	0	0	0	0
G4	Mean	2.00E-134	3.41E-132	8.15E-130	4.88E-127	4.79E-132	1.13E-128	3.87E-129	1.60E-132
	Std	9.87E-134	1.59E-131	3.77E-129	2.19E-126	1.62E-131	4.55E-128	1.38E-128	4.65E-132
G5	Mean	0	0	0	0	0	0	0	0
	Std	0	0	0	0	0	0	0	0
G6	Mean	1.74E-31	4.75E-26	3.58E-19	2.01E-14	1.10E-25	1.72E-17	2.69E-18	1.08E-25
	Std	4.17E-31	9.13E-26	5.58E-19	4.22E-14	2.23E-25	4.91E-17	6.37E-18	2.65E-25
G7	Mean	1.16E-05	1.44E-05	1.61E-05	1.79E-05	1.43E-05	1.72E-05	1.36E-05	1.59E-05
	Std	9.49E-06	1.21E-05	1.05E-05	1.40E-05	1.48E-05	1.44E-05	1.05E-05	1.23E-05
G8	Mean	0	0	0	0	0	0	0	0
	Std	0	0	0	0	0	0	0	0
G9	Mean	8.87E-16	8.87E-16	8.87E-16	8.87E-16	8.87E-16	8.87E-16	8.87E-16	8.87E-16
	Std	0	0	0	0	0	0	0	0
G10	Mean	0	0	0	0	0	0	0	0
	Std	0	0	0	0	0	0	0	0
G11	Mean	1.18E-22	5.76E-19	9.08E-15	3.47E-12	5.25E-19	3.11E-14	1.34E-15	3.86E-19
	Std	2.83E-22	1.76E-18	3.46E-14	5.57E-12	1.25E-18	5.27E-14	3.37E-15	8.12E-19
G12	Mean	5.48E-28	3.06E-23	4.58E-18	1.09E-13	6.00E-23	1.20E-16	1.43E-17	3.88E-23
	Std	1.60E-27	9.52E-23	8.95E-18	1.85E-13	1.92E-22	2.61E-16	2.67E-17	9.04E-23

Simulation results for different cases of NMRA breeding factor parameter $(\lambda )$									
Function		$\lambda _{0.25}$	$\lambda _{0.50}$	$\lambda _{0.75}$	$\lambda _{sa}$	$\lambda _{exp}$	$\lambda _{linear}$	$\lambda _{chaotic}$	$\lambda _{log}$
G1	Mean	3.76E-262	1.03E-263	1.46E-264	6.77E-263	7.62E-263	2.85E-263	8.09E-259	1.92E-263
	Std	0	0	0	0	0	0	0	0
G2	Mean	1.13E-132	6.77E-132	2.69E-132	5.49E-132	3.17E-132	1.21E-132	6.68E-132	2.25E-132
	Std	4.42E-132	4.43E-131	7.94E-132	1.93E-131	9.89E-132	2.72E-132	2.86E-131	7.00E-132
G3	Mean	7.34E-264	1.34E-261	6.41E-264	2.40E-264	2.68E-263	2.38E-262	1.36E-264	8.60E-263
	Std	0	0	0	0	0	0	0	0
G4	Mean	5.11E-132	1.12E-132	4.88E-132	3.93E-132	8.25E-132	1.09E-132	3.79E-132	8.06E-133
	Std	2.82E-131	3.53E-132	2.95E-131	2.16E-131	4.26E-131	3.87E-132	1.46E-131	2.58E-132
G5	Mean	0	0	0	0	0	0	0	0
	Std	0	0	0	0	0	0	0	0
G6	Mean	2.66E-25	1.42E-25	3.88E-25	2.66E-25	5.96E-26	1.64E-25	8.88E-26	9.14E-26
	Std	7.55E-25	3.75E-25	9.40E-25	1.21E-24	1.18E-25	5.87E-25	2.94E-25	2.60E-25
G7	Mean	1.81E-05	1.79E-05	1.81E-05	1.68E-05	1.59E-05	1.68E-05	1.49E-05	1.46E-05
	Std	1.98E-05	1.25E-05	1.52E-05	1.49E-05	1.64E-05	1.08E-05	1.10E-05	1.48E-05
G8	Mean	0	0	0	0	0	0	0	0
	Std	0	0	0	0	0	0	0	0
G9	Mean	8.87E-16	8.87E-16	8.87E-16	8.87E-16	8.87E-16	8.87E-16	8.87E-16	8.87E-16
	Std	0	0	0	0	0	0	0	0
G10	Mean	0	0	0	0	0	0	0	0
	Std	0	0	0	0	0	0	0	0
G11	Mean	5.33E-19	5.61E-19	4.62E-19	1.43E-18	1.60E-18	5.47E-19	3.07E-19	1.57E-18
	Std	1.91E-18	1.53E-18	9.31E-19	2.74E-18	9.21E-18	1.16E-18	7.38E-19	6.12E-18
G12	Mean	6.76E-23	5.83E-23	7.83E-23	2.88E-23	6.97E-23	1.03E-22	4.53E-23	6.17E-23
	Std	2.12E-22	1.06E-22	2.84E-22	7.66E-23	1.94E-22	3.79E-22	1.04E-22	2.30E-22

### Dimension size analysis

**Fig. 3:**
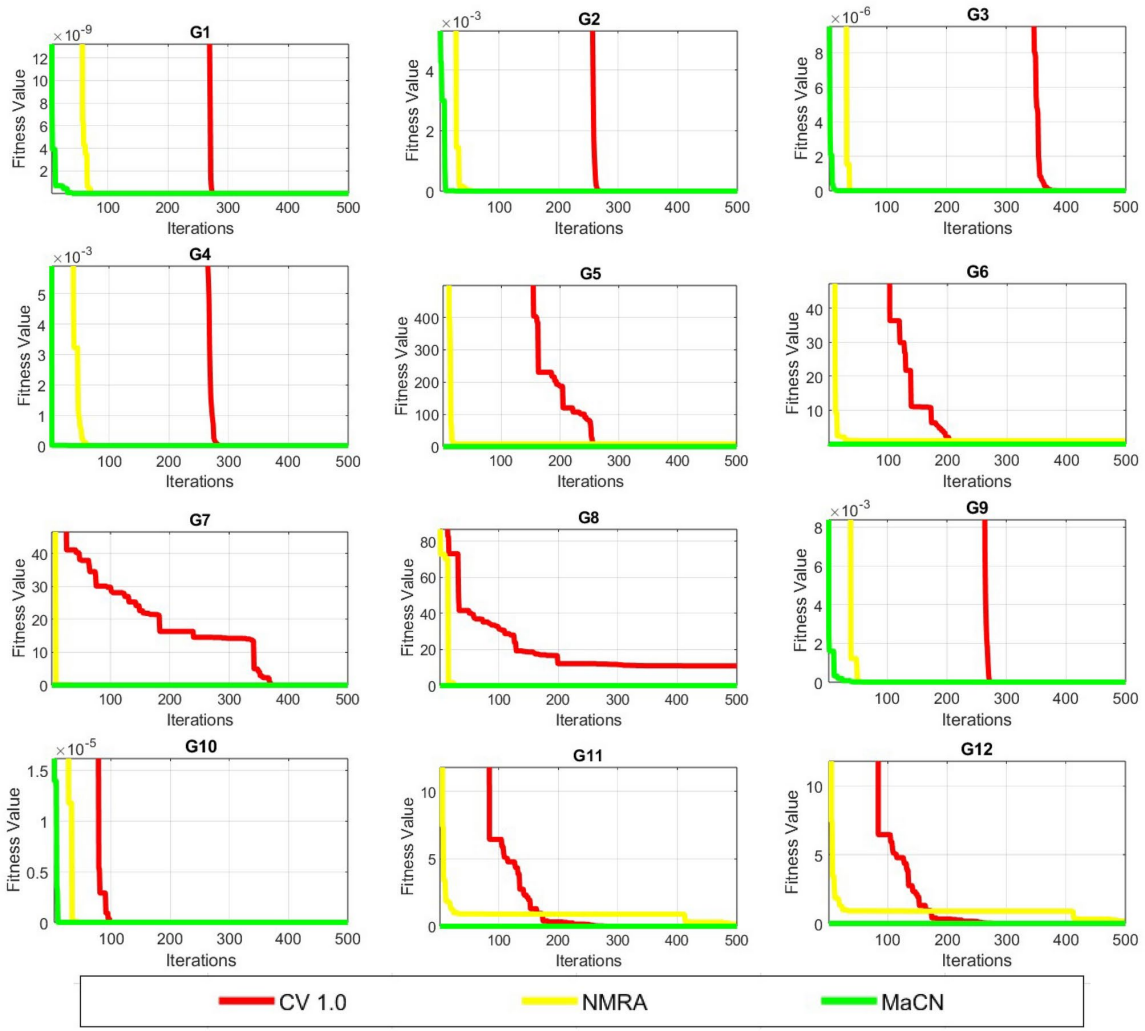
Convergence profiles of CV 1.0, NMRA and MaCN for Dimension Size 10.

The problem’s dimension size (*Dim*) is important for evaluating the efficiency of any optimization procedure. The performance of MaCN with respect to CV 1.0 and NMRA is examined in this subsection for 5 dimension sizes (10, 50, 100, 200, and 500). Dimensional analysis was used to see if MaCN might provide an optimal solution of objective functions for higher dimensions (CEC 2005). The statistical findings for all sets of dimension sizes are in Table 5 as mean and *std* of 51 runs and 500 iterations. The following is a full study of these results for various dimension sizes:

*Dimension size 10:* The simulation results for $Dim = 10$ are shown in Table 5, and it was discovered that only MaCN delivers a solution close to the optimal value for *G*1, *G*2, *G*3, and *G*4. The MaCN results are zero and the same as the global minimal value in *G*5 and *G*8. Compared to CV 1.0 and NMRA, MaCN is more efficient for *G*6, *G*7, *G*11, and *G*12. Finally, NMRA and MaCN show the same performance for *G*9 and *G*10, making it competitive to choose the optimal algorithm for these functions. Apart from simulated results, convergence profiles are also drawn and shown in Figure 3. As a result, MaCN is considered the best algorithm in this scenario.

**Fig. 4:**
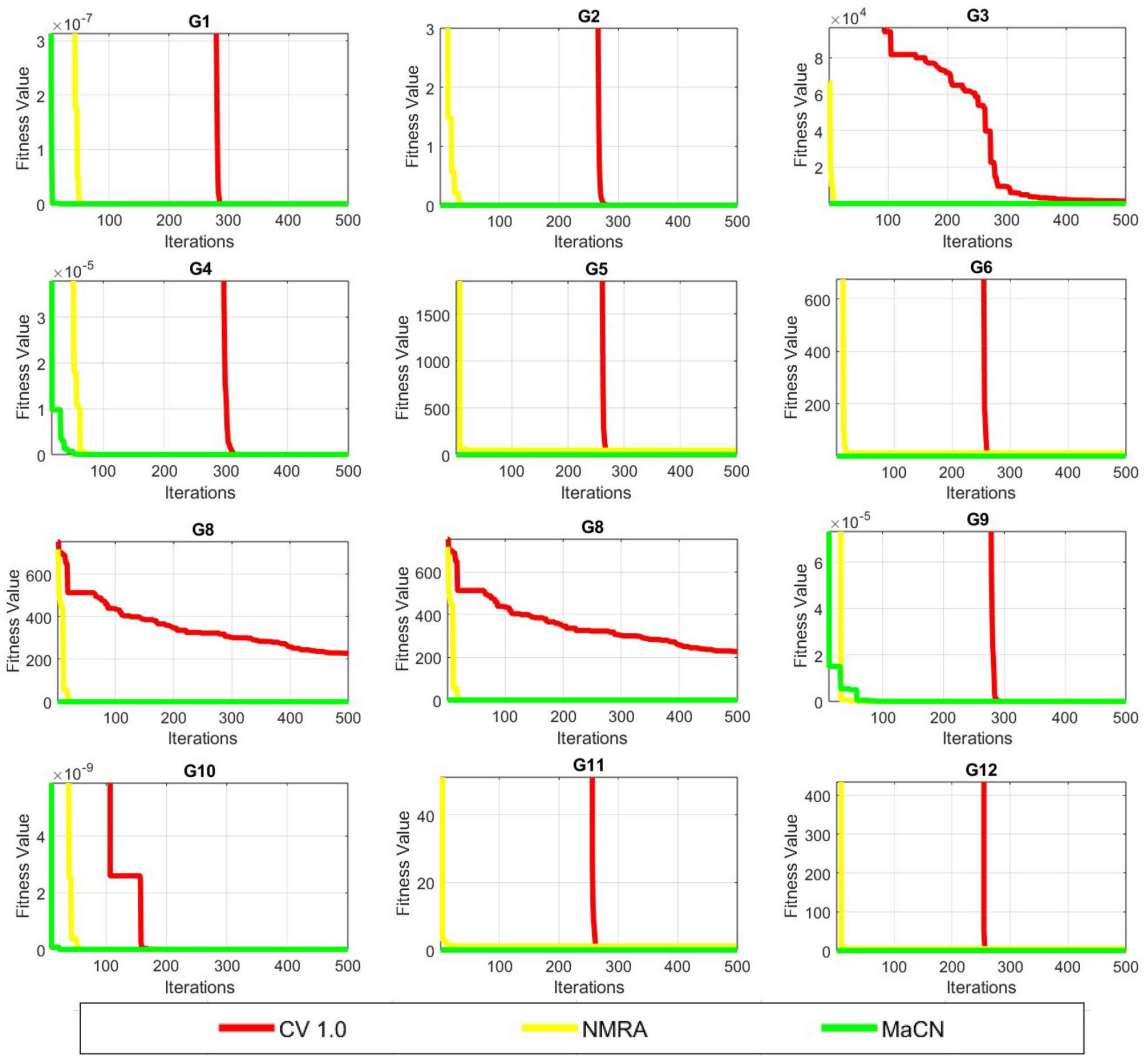
Convergence profiles of CV 1.0, NMRA and MaCN for Dimension Size 50.

*Dimension Size 50:* Table 5 displays the statistical results for $Dim = 50$. MaCN is the best algorithm for *G*1, *G*2, *G*3, and *G*4, and no other algorithm can match it. MaCN provides a global minimum solution for *G*5. Compared to NMRA and CV 1.0, MaCN produces better for *G*6, *G*7, *G*11, and *G*12. Finally, the NMRA and MaCN values for *G*8, *G*9, and *G*10 are identical, with a zero standard deviation value. Figure 4 shows the convergence profiles for this dimension size. As a result, the MaCN results are again found to be appropriate here.

**Fig. 5:**
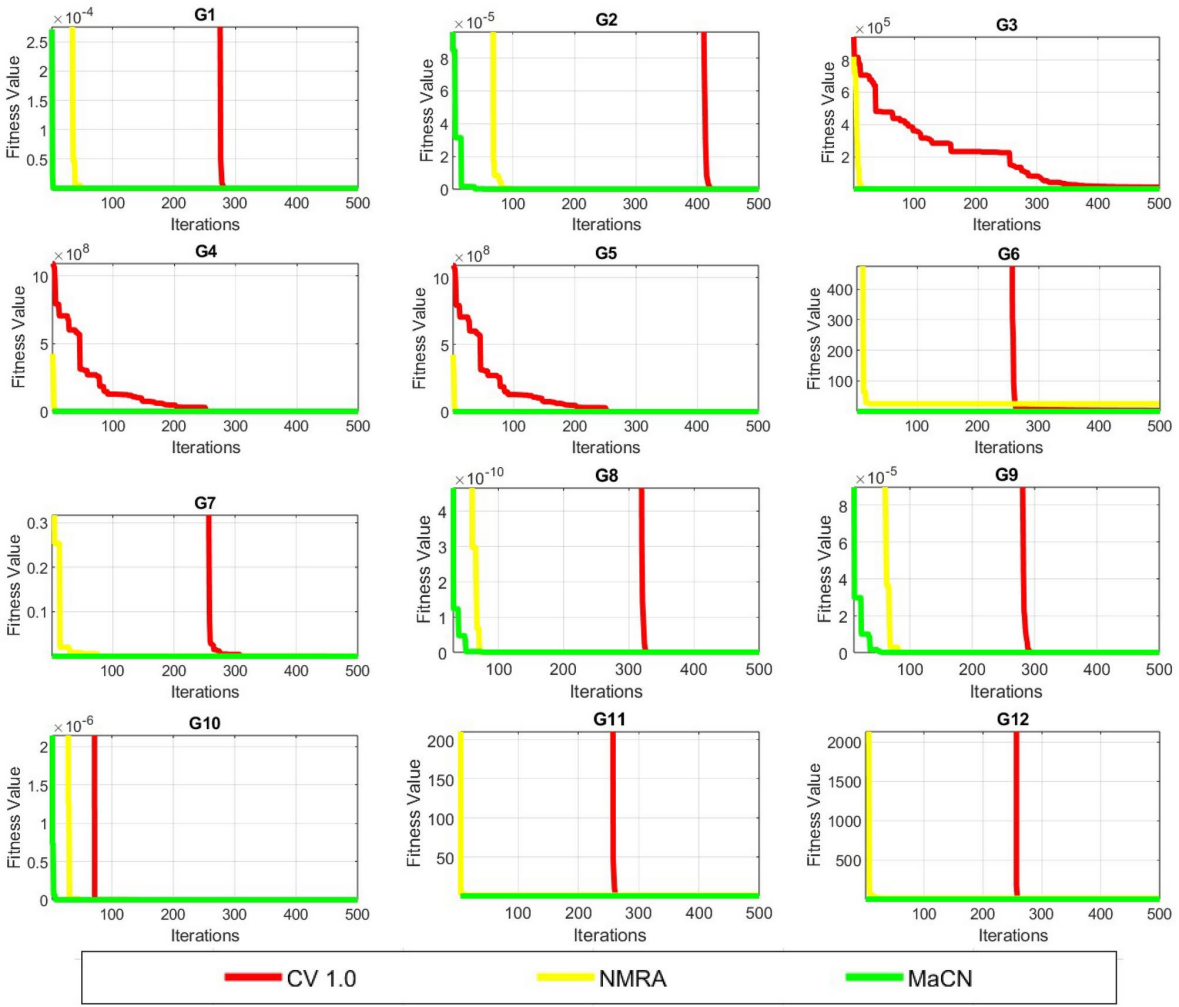
Convergence profiles of CV 1.0, NMRA and MaCN for Dimension Size 100.

*Dimension Size 100:* Table 5 shows the results for this set of dimension sizes, and MaCN obtains mean values that are close to the ideal for *G*1 and *G*3. The MaCN results for functions *G*2 and *G*4 are practically identical. MaCN provides the minimal global solution for *G*5. Compared to NMRA and CV 1.0, MaCN performs much better for *G*6, *G*7, *G*11, and *G*12. Both NMRA and MaCN have zero results for *G*8 and *G*10. MaCN and NMRA results for the last function *G*9 are near-global minima values (i.e.0). As a result, MaCN is once again the best choice for $Dim = 100$ and convergence profiles shown in Figure 5 validate it.

*Dimension Size 200:* Table 5 shows the simulated results for $Dim = 200$, and here results by MaCN are exactly zero and the same as the optimal value of the functions for *G*1, *G*3, and *G*5. When compared to CV 1.0 and NMRA, MaCN is more capable for *G*2 and *G*4. The MaCN method is determined as best for *G*6, *G*7, *G*11, and *G*12. Both MaCN and NMRA idential solutions for *G*8 and *G*10. Both MaCN and NMRA produce the best and similar outcomes in the last for *G*9. Overall, MaCN outperformed the competition in this scenario.

*Dimension Size 500:* Table 5 shows the results for $Dim= 500$ and shows that MaCN finds the best results for *G*1, *G*3, and *G*5. MaCN has the best performance in the *G*2 and *G*4 cases. Only MaCN can approach optimum outcomes for *G*6, *G*7, *G*11, and *G*12. The findings of MaCN, CV 1.0, and NMRA for *G*8 and *G*10 are identical and same as global optimal value. Finally, MaCN and NMRA values for *G*9 are identical and better than CV 1.0. As a result, MaCN is thought to be the best method for solving higher-dimensional problems.

*Inferences:* From the results for lower dimension, the algorithm shows significant performance. But as the dimension size increases, the results degrade due to increase in the computational complexity. Overall, it can be said that with increase in dimension size, the results do vary a little but the overall performance do not degrade much.

**Table 5:** Comparison of CV 1.0, NMRA and MaCN for five dimension sizes (10, 50, 100, 200 and 500).

Function	Algorithm	Dim = 10		Dim = 50		Dim = 100		Dim = 200		Dim Size 500	
		Mean	Std	Mean	Std	Mean	Std	Mean	Std	Mean	Std
G1	CV 1.0	7.81E-108	5.55E-107	7.64E-87	4.88E-86	1.88E-79	1.34E-78	4.61E-79	2.15E-78	1.05E-76	7.03E-76
	NMRA	1.56E-83	1.09E-82	1.48E-85	1.05E-84	1.04E-85	5.85E-85	1.64E-83	1.03E-83	9.23E-86	4.70E-84
	MaCN	4.45E-257	0	5.12E-271	0	2.01E-289	0	0	0	0	0
G2	CV 1.0	2.57E-59	1.48E-58	1.16E-18	8.32E-18	1.96E+08	1.40E+09	3.92E+08	1.96E+09	1.37E+09	3.47E+09
	NMRA	2.49E-46	1.02E-45	7.75E-45	3.58E-44	1.71E-43	8.03E-43	2.60E-44	1.54E-43	4.25E-44	3.00E-43
	MaCN	1.22E-128	3.23E-128	2.17E-129	6.66E-129	1.15E-146	3.46E-146	1.22E-199	0	2.74E-293	0
G3	CV 1.0	5.22E-14	1.94E-13	1.50E+03	5.87E+02	9.48E+03	1.71E+03	4.06E+04	8.12E+03	3.09E+05	5.20E+04
	NMRA	8.28E-87	4.71E-86	1.00E-87	4.80E-87	9.14E-86	6.33E-85	7.13E-86	3.96E-85	5.42E-82	3.86E-81
	MaCN	3.32E-257	0	4.46E-254	0	1.02E-288	0	0	0	0	0
G4	CV 1.0	2.72E-45	1.76E-44	1.22E-33	6.45E-33	8.75E-30	6.15E-29	1.39E-28	4.58E-28	5.62E-24	2.80E-23
	NMRA	3.50E-45	1.08E-44	2.06E-44	6.86E-44	2.31E-44	1.37E-43	3.01E-45	9.54E-45	5.83E-45	2.12E-44
	MaCN	2.37E-128	9.74E-128	3.21E-130	7.84E-130	1.91E-146	1.04E-145	4.35E-199	0	2.37E-292	0
G5	CV 1.0	1.13E+00	1.17E+00	4.64E+01	6.86E-01	9.70E+01	5.85E-01	1.97E+02	4.60E-01	4.95E+02	3.16E-01
	NMRA	8.96E+00	1.81E-02	4.89E+01	2.05E-02	9.89E+01	2.55E-02	1.98E+02	2.77E-02	4.97E+02	1.81E-02
	MaCN	0	0	0	0	0	0	0	0	0	0
G6	CV 1.0	5.75E-07	2.76E-07	4.63E-01	2.07E-01	2.97E+00	6.80E-01	1.08E+01	1.89E+00	4.61E+01	6.18E+00
	NMRA	1.40E+00	6.96E-01	1.16E+01	6.91E-01	2.42E+01	5.03E-01	4.91E+01	7.11E-01	1.24E+02	5.92E-01
	MaCN	7.82E-22	1.80E-21	8.26E-13	1.52E-12	1.84E-14	2.60E-14	2.41E-14	4.74E-14	6.50E-28	1.20E-27
G7	CV 1.0	5.75E-04	4.60E-04	7.92E-04	6.06E-04	6.95E-04	5.45E-04	9.83E-04	7.55E-04	9.32E-04	6.28E-04
	NMRA	8.09E-04	8.34E-04	5.21E-04	4.67E-04	6.43E-04	5.83E-04	5.98E-04	5.72E-04	8.32E-04	8.74E-04
	MaCN	4.12E-05	3.25E-05	1.23E-05	9.90E-06	5.57E-06	4.76E-06	3.40E-06	2.74E-06	2.26E-06	1.41E-06
G8	CV 1.0	4.64E+00	5.07E+00	1.80E+02	1.25E+02	8.27E+01	2.29E+02	3.92E-02	2.79E-01	0	0
	NMRA	5.22E-01	3.73E+00	0	0	0	0	0	0	0	0
	MaCN	0	0	0	0	0	0	0	0	0	0
G9	CV 1.0	1.02E-15	6.96E-16	1.44E-15	1.30E-15	1.72E-15	1.52E-15	1.72E-15	1.52E-15	2.00E-15	1.66E-15
	NMRA	8.87E-16	0	8.87E-16	0	8.87E-16	0	8.87E-16	0	8.87E-16	0
	MaCN	8.87E-16	0	8.87E-16	0	8.87E-16	0	8.87E-16	0	8.87E-16	0
G10	CV 1.0	0	0	0	0	0	0	0	0	0	0
	NMRA	0	0	0	0	0	0	0	0	0	0
	MaCN	0	0	0	0	0	0	0	0	0	0
G11	CV 1.0	4.61E-07	2.94E-07	1.06E-02	4.00E-03	2.81E-02	7.70E-03	5.74E-02	1.79E-02	1.20E-01	2.52E-02
	NMRA	6.77E-01	3.41E-01	1.16E+00	1.51E-01	1.19E+00	8.62E-02	1.19E+00	4.54E-02	1.17E+00	1.51E-02
	MaCN	1.82E-16	3.10E-16	9.74E-11	1.99E-10	1.60E-12	3.05E-12	2.87E-12	4.49E-12	3.32E-21	5.25E-21
G12	CV 1.0	8.63E-04	3.00E-03	5.30E-01	2.12E-01	2.01E+00	5.18E-01	6.48E+00	1.51E+00	1.36E+09	3.46E+09
	NMRA	8.83E-01	1.93E-01	4.98E+00	2.90E-03	9.99E+00	3.00E-03	1.99E+01	2.20E-03	4.99E+01	2.50E-03
	MaCN	1.68E-19	6.47E-19	3.15E-11	7.37E-11	7.97E-13	8.14E-12	1.18E-12	2.44E-12	1.05E-25	1.96E-25

### Performance evaluation for CEC 2005 test suite in comparsion with other algorithms

The capability of proposed MaCN algorithm is tested in this subsection by doing comparison with various algorithms such as SHADE, FA-FPO, JADE, GWO-E and others. These algorithms are found to be very competitive and capable in solving complex problems of optimization. The selection of parameter values is provided in Table 3. Here, CEC 2005 test functions are used as benchmark for evaluating performance of MaCN with $Dim = 30$, and definition of these test functions are provided in Table 2.

#### Experimental testing

Table 6 contains results for all the algorithms. Only MaCN can give near-optimal global solutions for *G*1, *G*2, *G*3, and *G*4, as shown by the findings. MaCN achieves global minima for *G*5, while all others couldnot. MaCN outperforms *G*6 in terms of mean and std. The results of FA-FPO and MaCN in the situation of *G*7 are fairly competitive, but MaCN is the best in this scenario. FA-FPO, GWO-E, OEWOA, EO, MGSCA, and suggested MaCN all produce comparable and ideal outcomes for *G*8. In the instance of *G*9, FA-FPO and MaCN function equally well. GWO-E, FA-FPO, EO, and MaCN obtain the best global value for *G*10. Finally, MaCN outperforms others for *G*11 and *G*12. MaCN is optimal for all functions, FA-FPO for three, OEWOA and MGSCA for one, and EO and GWO-E for two, according to the 12 test functions.

#### Statistical testing

Here wilcoxon’s rank-sum test^139^and Friedman rank (f-rank)^47^, are used to statistically validate the MaCN algorithm’s simulated findings. These tests helps to find the statistical significance of an algorithm and are described as:

*Wilcoxon’s ran-sum test:* This test uses a *win*(*w*)/*loss*(*l*)/*tie*(*t*) stategy to find the best algorithm under consideration. A p-rank is assigned to each of the algorithm and the algorithm with the lowest rank is considered as the winner. Here $win(w) = +$ means that the algorithm outperforms the proposed MaCN, $loss(l) = -$ means MaCN outperforms the test algorithm, and $tie(t) = =$ means both have the similar performance. The results for this test is given in Table 6, and MaCN scores the best among all in majority of the cases.

*Friedman rank test:* This test gives a rank (*f-rank*) to each of the algorithm ranging from 1 to the maximum number of algorithms under test. The algorithm with the least rank is treated as the best. Table 6 shows that among all the cases, the MaCN algorithm scores the first rank for the average and overall *f-rank*.

**Table 6:** Simulated results for CEC 2005 test suite.

Function		JADE	SaDE	GWO-E	OEWOA	SCCS	FA-FPO	CMA-ES	SHADE	LSHADE-	EO	BDE	MGSCA	MaCN
		^38^	^38^	^40^	^42^	^37^	^44^	^35^	^35^	SPACMA^35^	^35^	^33^	^33^	
*G*1	mean	1.80E-60	4.50E-20	3.92E-67	7.75E-176	9.22E-69	1.51E-184	1.42E-18	1.42E-09	2.23E-01	3.32E-40	3.86E-02	1.17E-104	**2.52E-262**
	std	8.40E-60	6.90E-20	1.11E-66	0	3.81E-68	0	3.13E-18	3.09E-09	1.48E-01	6.78E-40	1.97E-01	6.08E-104	**0**
	p-rank	−	−	−	−	−	−	−	−	−	−	−	−	
	f-rank	7	9	6	3	5	2	10	11	13	8	12	4	1
*G*2	mean	1.80E-25	1.90E-14	4.31E-36	1.86E-115	8.25E-41	5.04E-93	2.98E-07	8.70E-03	2.11E+01	7.12E-23	6.97E-01	5.80E-68	**9.00E-132**
	std	8..8E-25	1.05E-14	6.57E-36	1.32E-114	4.19E-40	3.47E-93	1.78E+00	2.13E-02	9.57E+00	6.36E-23	3.34E+00	1.34E-67	**3.42E-131**
	p-rank	−	−	−	−	−	−	−	−	−	−	−	−	
	f-rank	7	9	6	2	5	3	10	11	13	8	12	4	1
*G*3	mean	5.70E-61	9.00E-37	3.75E-37	2.87E+04	4.31E-13	1.23E-183	1.59E-05	1.54E+01	8.87E+01	8.06E-09	1.80E+03	7.92E-20	**1.34E-262**
	std	2.70E-60	5.43E-36	1.36E-36	1.02E+04	2.83E-30	0	2.21E-05	9.94E+00	4.72E+01	1.60E-08	2.88E+03	2.68E-19	**0**
	p-rank	−	−	−	−	−	−	−	−	−	−	−	−	
	f-rank	3	5	4	13	7	2	9	10	11	8	12	6	1
*G*4	mean	8.20E-24	7.40E-11	2.39E-25	1.06E+01	2.15E-17	9.97E-93	2.01E-06	9.79E-01	2.11E+00	5.39E-10	4.80E+01	6.79E-14	**6.92E-133**
	std	4.00E-23	1.82E-10	6.80E-25	2.22E+01	1.06E-16	7.31E-93	1.25E-06	7.99E-01	4.92E-01	1.38E-09	1.09E+01	2.08E-13	**2.09E-132**
	p-rank	−	−	−	−	−	−	−	−	−	−	−	−	
	f-rank	4	7	3	12	5	2	9	10	11	8	13	6	1
*G*5	mean	8.00E-02	2.10E+01	2.65E+01	2.85E+01	5.90E+00	2.89E+01	3.67E+01	2.44E+01	2.88E+01	2.53E+01	2.11E+04	2.68E+01	**0**
	std	5.60E-01	7.80E+00	5.19E-01	2.22E-01	9.13E-01	1.72E-02	3.34E+01	1.12E+01	8.24E-01	1.69E-01	1.06E+05	9.70E-01	**0**
	p-rank	−	−	−	−	−	−	−	−	−	−	−	−	
	f-rank	2	4	7	9	3	11	12	5	10	6	13	8	1
*G*6	mean	2.90E+00	9.30E+02	2.65E+01	1.62E+00	4.14E-08	5.88E+00	6.83E-19	5.31E-10	2.48E-01	8.29E-06	4.37E+01	1.34E+00	**9.55E-26**
	std	1.20E+00	1.80E+02	5.19E-01	6.93E-01	5.22E-08	5.86E-01	6.71E-19	6.35E-10	1.13E-01	5.02E-06	2.38E+02	4.67E-01	**3.55E-25**
	p-rank	−	−	−	−	−	−	−	−	−	−	−	−	
	f-rank	9	13	11	8	4	10	2	3	6	5	12	7	1
*G*7	mean	6.40E-04	4.80E-03	9.90E-04	1.37E-03	1.33E-03	1.13E-04	2.75E-02	2.35E-02	4.70E-03	1.17E-03	3.68E-01	1.77E-03	**1.83E-05**
	std	2.50E-04	1.20E-03	8.37E-04	2.85E-03	1.72E-03	8.94E-04	7.90E-03	8.80E-03	1.90E-03	6.54E-04	9.56E-01	1.33E-03	**1.78E-05**
	p-rank	−	−	−	−	−	−	−	−	−	−	−	−	
	f-rank	3	10	4	7	6	2	12	11	9	5	13	8	1
*G*8	mean	1.00E-04	1.20E-03	**0**	**0**	5.46E+00	**0**	2.53E+01	8.53E+00	6.75E+01	**0**	5.50E+01	**0**	**0**
	std	6.00E-05	6.50E-04	**0**	**0**	5.62E+00	**0**	8.55E+00	2.19E+00	1.00E+01	**0**	3.26E+01	**0**	**0**
	p-rank	−	−	$=$	$=$	−	$=$	−	−	−	$=$	−	$=$	
	f-rank	7	8	1	1	9	1	11	10	13	1	12	1	1
*G*9	mean	8.20E-10	2.70E-03	5.58E-15	3.02E-15	**8.88E-16**	**8.88E-16**	1.55E+01	3.95E-01	3.93E-02	8.34E-14	1.67E+01	8.69E+00	**8.88E-16**
	std	6.90E-10	5.10E-04	1.67E-15	2.27E-15	9.36E-32	**0**	7.92E+00	5.86E-01	1.51E-02	2.53E-14	6.08E+00	1.01E+01	**0**
	p-rank	−	−	−	−	−	$=$	−	−	−	−	−	−	
	f-rank	7	8	5	4	3	1	12	10	9	6	13	8	1
*G*10	mean	9.90E-08	7.80E-04	**0**	1.42E-02	3.33E-02	**0**	5.76E-15	4.80E-03	8.94E-01	**0**	4.83E-01	3.21E-03	**0**
	std	6.00E-07	1.20E-03	**0**	1.00E-01	4.56E-02	**0**	6.18E-15	7.70E-03	1.07E-01	**0**	1.40E+00	8.51E-03	**0**
	p-rank	−	−	$=$	−	−	$=$	−	−	−	$=$	−	−	
	f-rank	6	7	1	10	11	1	5	9	13	1	12	8	1
*G*11	mean	4.60E-17	1.90E-05	1.98E-02	1.06E-01	1.34E-02	8.32E-01	2.87E-16	3.46E-02	8.18E-04	7.97E-07	8.87E+04	7.07E-02	**3.06E-19**
	std	1.90E-16	9.20E-06	1.01E-02	4.97E-02	1.60E-02	1.78E-01	5.64E-16	8.75E-02	1.00E-03	7.69E-07	1.88E+05	3.16E-02	**6.55E-19**
	p-rank	−	−	−	−	−	−	−	−	−	−	−	−	
	f-rank	2	5	8	11	7	12	3	9	6	4	13	10	1
*G*12	mean	2.00E-16	6.10E-05	2.50E-01	1.03E+00	2.01E-02	2.94E+00	3.66E-04	7.32E-04	1.02E-02	2.92E-02	4.05E+05	1.42E+00	**2.06E-23**
	std	6.50E-16	2.00E-05	1.63E-01	3.61E-01	7.23E-02	1.59E-01	2.00E-03	2.80E-03	1.03E-02	3.52E-02	1.33E+06	2.57E-01	**6.52E-23**
	p-rank	−	−	−	−	−	−	−	−	−	−	−	−	
	f-rank	2	3	9	10	7	13	4	5	6	8	13	11	1
w/l/t		0/12/0	0/12/0	0/10/2	0/11/1	0/12/0	0/9/3	0/12/0	0/12/0	0/12/0	0/10/2	0/12/0	0/11/1	NA
Average f-rank		4.92	7.33	5.41	7.50	6.00	5.00	8.25	8.67	10.00	5.67	12.50	7.00	1.00
Overall f-rank		2	8	4	9	6	3	10	11	12	5	13	7	1

#### Convergence profiles and box-plots

**Fig. 6:**
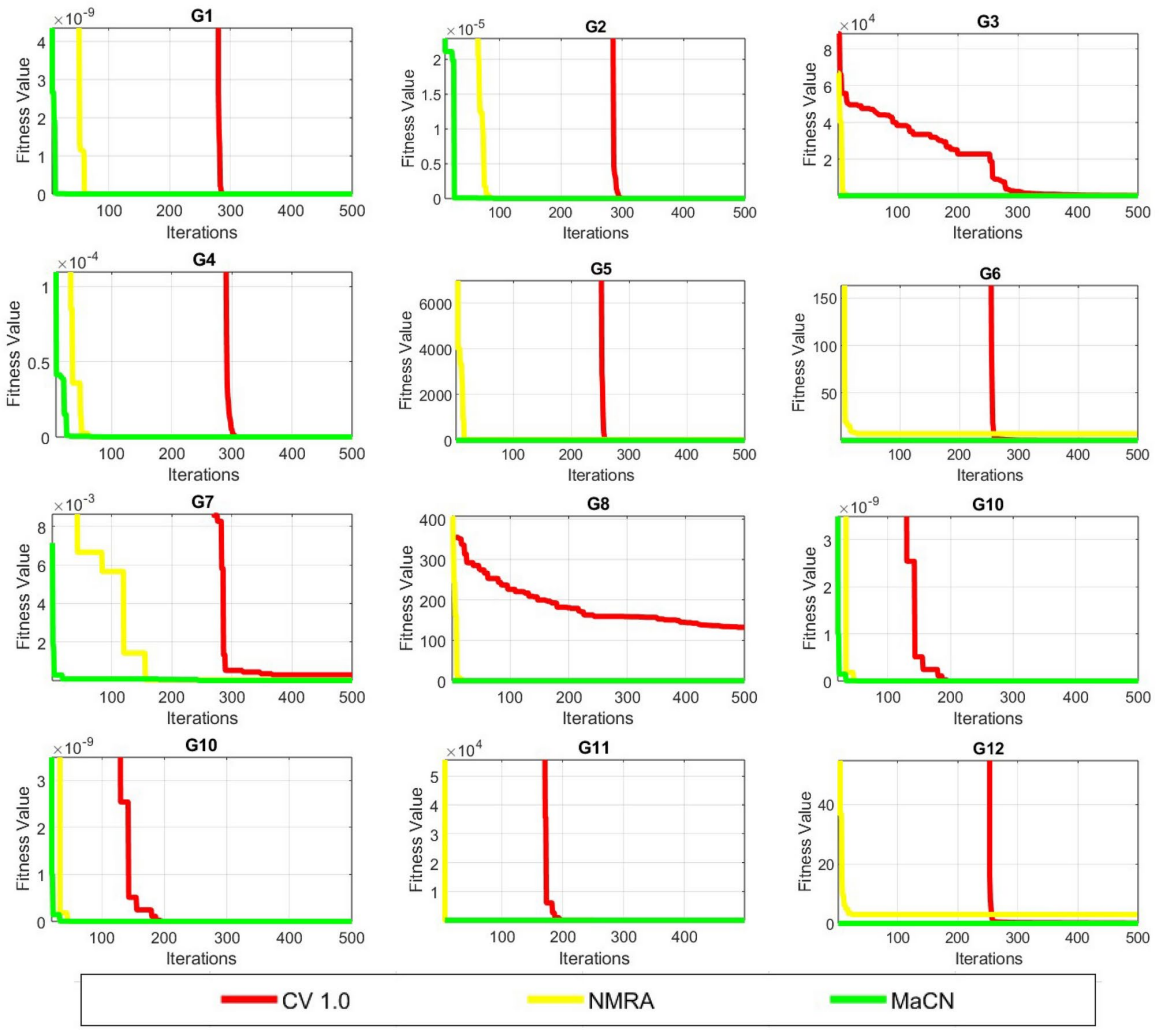
Convergence profiles of CV 1.0, NMRA and MaCN for Dimension Size 30.

**Fig. 7:**
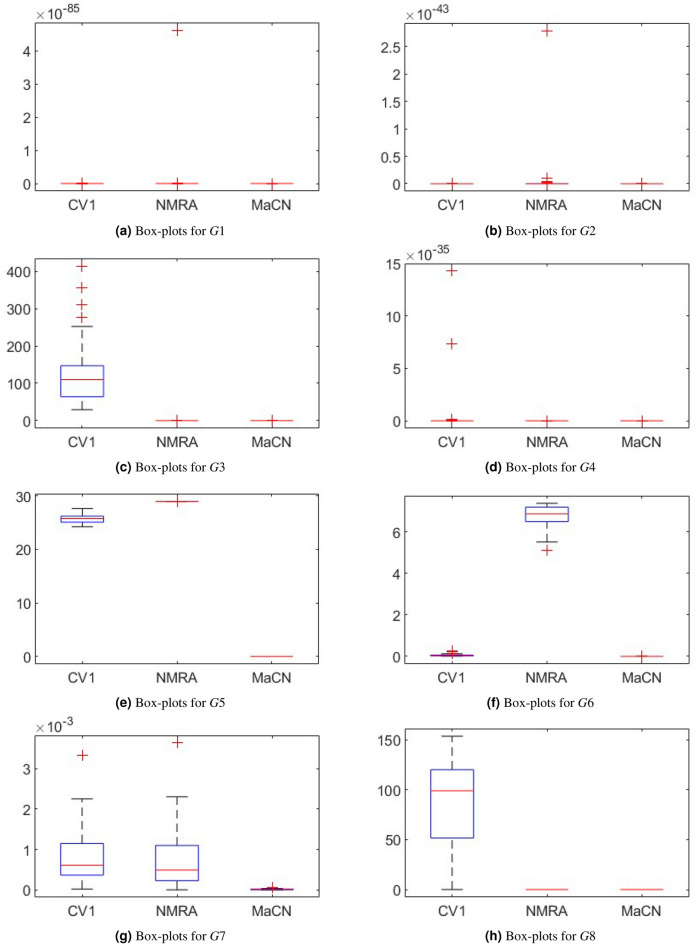

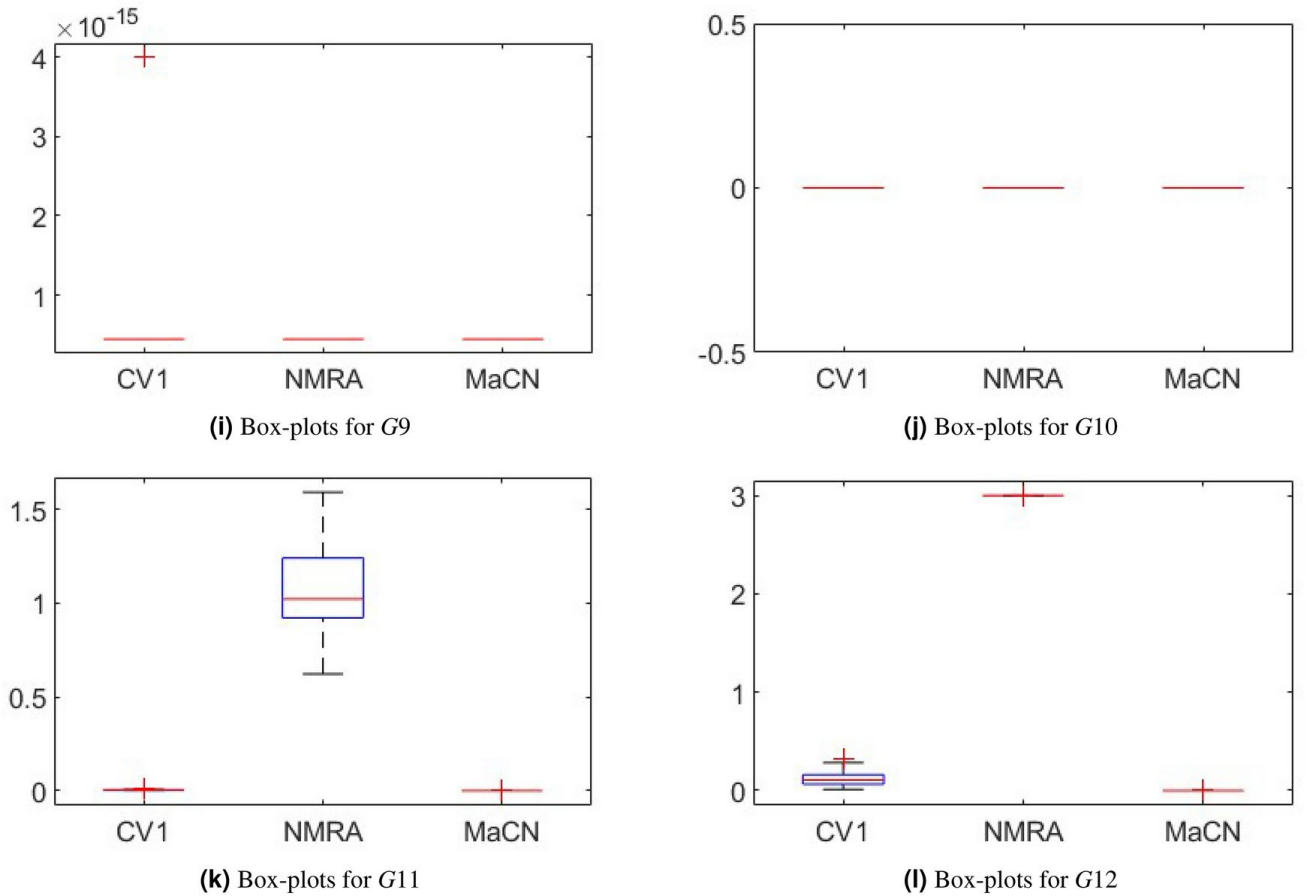
Box-plots of CV1, NMRA and MaCN for CEC 2005 test suite.

To analyze the convergence behavior of CV 1.0, NMRA, and proposed MaCN, the convergence graphs are drawn for 7 uni-modal (*G*1 to *G*7)and 5 multi-modal (*G*8 to *G*12) functions of CEC 2005 benchmark suite as shown in Figure 6. From this figure, we see that MaCN converges faster as compared to CV 1.0 and NMRA for *G*1, *G*3, *G*4, *G*9, *G*10, *G*11 and *G*12. For *G*2, NMRA converges during the initial iterations, but MaCN and CV 1.0 converge later. For *G*5, *G*7, and *G*8, the convergence of MaCN and NMRA are almost similar while CV 1.0 converges at a slower rate during iterations. For function *G*6, MaCN attains a global minimal value in the early stages, but NMRA cannot converge towards the end. So, overall, it can be said that MaCN shows better convergence in most cases than CV 1.0 and NMRA. In addition to convergence profiles, Figure 7 presents box-plots that compare the fitness values of CV1, NMRA and the proposed MaCN. The results demonstrate that MaCN is more cost-effective in terms of fitness, as evidenced by its lower median fitness value compared to the other algorithms.

### Comparison of proposed approach for CEC2014 test suite

To prove the effectiveness of proposed MaCN algorithm, it is further tested on challenging CEC 2014 test problems^140^and definition of these problems are available in^132^. For these benchmark functions, total function evaluations are set to $10^4$ $\times$ *Dim*^140^. Here, performance of MaCN is compared with different improved meta-heuristic algorithms such as VNBA, RW-GWO, LX-BBO, MGSCA and others. The parameter selection of all the algorithms under comparison are listed in Table 3.

MaCN’s statistical findings for CEC 2014 benchmark issues are reported as mean error and *std* values for 51 independent algorithm runs with $Dim = 30$. Refer to 7 for a summary of these findings. ISOS performs well in the $F_1$, $F_2$, and $F_{24}$ categories, according to this table. MaCN’s results are close to optimal in the instance of $F_3$. RW-GWO and MaCN both have good results for $F_4$, but RW-GWO is the best overall. LX-BBO has the best performance for $F_5$ based on mean error values. MaCN is found to be the best given only mean values for $F_6$, $F_{10}$, $F_{11}$, and $F_{16}$. IMEHO’s mean error value is the same as optimum value for $F_7$, $F_{13}$, $F_{14}$, and $F_{15}$. B-BBO outperforms its competitors in $F_8$. IMEHO produces good results in the cases of $F_9$ and $F_{26}$. B-BBO and LX-BBO are the best and identical outcomes for $F_{12}$. The suggested algorithm performs well for $F_{17}$, $F_{18}$, $F_{19}$, $F_{20}$, $F_{21}$, $F_{22}$, $F_{23}$, and $F_{30}$ in both mean error and *std* values. Finally, MaCN can reach global minima for *std* values for $F_{25}$, $F_{27}$, $F_{28}$, and $F_{29}$. Thus, ISOS outperforms RW-GWO for three functions, IMEHO for six, B-BBO and LX-BBO for two functions, and proposes MaCN for seventeen functions from the 30 benchmark tasks of the CEC 2014 test suite. The findings of various algorithms are also validated using the f-rank statistical test. After assigning a rank to each method under test, the average and overall f-rank are computed and displayed in Table 7. It can be seen that MaCN is ranked first and outperforms all of the competing algorithms.

**Table 7:** Results of MaCN algorithm for CEC 2014 test functions.

Functions		LX-BBO^50^	B-BBO^50^	RW-GWO^48^	ISOS^47^	PBIL^52^	VNBA^52^	IMEHO^52^	CCS^52^	BDE^33^	MGSCA^33^	MaCN
$F_1$	mean	1.01E+07	6.51E+06	8.02E+06	**9.82E+05**	3.42E+08	2.43E+08	2.37E+06	1.46E+08	1.25E+07	2.92E+07	1.18E+06
	std	1.01E+07	1.31E+06	3.32E+06	7.05E+05	1.09E+08	5.93E+07	4.32E+06	3.27E+07	7.47E+03	2.07E+07	**5.51E+05**
	f-rank	6	4	5	1	11	10	3	9	7	8	2
$F_2$	mean	5.34E+04	2.35E+04	2.23E+05	**5.27E+00**	4.08E+10	1.92E+10	5.49E+03	2.60E+09	9.25E+03	2.26E+09	1.00E+10
	std	2.14E+04	9.99E+03	5.51E+05	1.72E+01	3.39E+09	4.23E+09	4.87E+03	5.22E+08	6.19E+04	1.69E+09	**0**
	f-rank	5	4	6	1	11	10	2	7	3	8	9
$F_3$	mean	1.63E+04	6.03E+03	3.16E+02	4.79E+02	9.18E+04	2.93E+04	1.40E+02	2.70E+03	5.27E+02	1.77E+04	**1.25E+01**
	std	1.70E+04	3.15E+03	4.34E+02	6.24E+02	1.75E+04	1.39E+04	1.58E+02	7.74E+04	1.11E+03	6.63E+03	**3.43E+00**
	f-rank	8	7	3	4	11	10	2	6	5	9	1
$F_4$	mean	9.99E+01	1.02E+02	**3.41E+01**	5.98E+01	3.42E+03	1.60E+03	1.24E+02	3.22E+02	5.76E+02	2.76E+02	5.85E+01
	std	2.84E+01	3.13E+01	**1.81E+01**	3.56E+01	7.56E+02	3.63E+02	4.77E+01	4.09E+01	4.29E+01	6.56E+01	2.63E+01
	f-rank	4	5	1	3	11	10	6	8	9	7	2
$F_5$	mean	**3.06E+00**	3.74E+00	2.05E+01	2.03E+01	2.10E+01	2.10E+01	2.10E+01	2.10E+01	2.01E+01	2.03E+01	2.02E+01
	std	7.85E-01	4.91E-01	7.46E-02	6.67E-02	5.56E-02	**5.43E-02**	5.99E-02	8.81E-02	1.33E-01	1.44E-01	2.58E-01
	f-rank	1	2	7	4	9	8	10	11	3	6	5
$F_6$	mean	1.70E+01	1.99E+01	9.83E+00	1.05E+01	3.81E+01	3.30E+01	1.21E+01	2.51E+01	2.38E+01	1.94E+01	**9.65E+00**
	std	3.12E+00	2.70E+00	3.49E+00	2.39E+00	**1.16E+00**	2.58E+00	2.72E+00	2.01E+00	4.39E+00	2.88E+00	1.88E+00
	f-rank	5	7	2	3	11	10	4	9	8	6	1
$F_7$	mean	1.75E-01	7.81E-02	2.51E-01	1.54E-02	3.41E+02	1.10E+02	**0**	2.31E+01	1.51E+00	1.99E+01	5.90E-03
	std	8.56E-02	4.43E-02	1.42E-01	1.83E-02	2.74E+01	1.81E+01	1.19E-01	3.52E+00	6.07E+00	1.18E+01	**6.30E-03**
	f-rank	5	4	6	3	11	10	1	9	7	8	2
$F_8$	mean	5.53E+01	**4.71E-01**	4.38E+01	1.47E+01	3.01E+02	1.74E+02	3.30E+01	2.90E+02	6.12E+01	1.07E+02	3.30E+01
	std	3.78E+02	**6.78E-01**	8.47E+00	3.34E+00	1.03E+01	1.61E+01	9.19E+00	2.23E+01	2.60E+01	2.14E+01	1.18E+01
	f-rank	6	1	5	2	11	9	3	10	7	8	4
$F_9$	mean	7.66E+01	9.10E+01	6.33E+01	2.54E+02	3.69E+02	2.50E+02	**3.20E+01**	2.90E+02	1.10E+02	1.39E+02	1.39E+02
	std	1.60E+01	1.54E+01	1.30E+01	1.33E+01	1.69E+01	2.04E+01	**1.15E+01**	2.38E+01	4.69E+01	2.60E+01	1.52E+01
	f-rank	3	4	2	9	11	8	1	10	5	7	6
$F_{10}$	mean	1.25E+04	6.68E+03	9.61E+02	1.78E+03	6.26E+03	3.50E+03	2.26E+03	8.55E+03	1.75E+03	2.82E+03	**6.65E+02**
	std	1.16E+02	4.58E+02	2.72E+02	**4.09E+01**	3.05E+02	3.47E+02	5.72E+02	4.91E+02	7.28E+02	6.83E+02	3.04E+02
	f-rank	11	9	2	4	8	7	5	10	3	6	1
$F_{11}$	mean	1.23E+04	6.71E+03	2.68E+03	1.48E+03	7.10E+03	6.80E+03	2.86E+03	8.83E+03	3.90E+03	3.30E+03	**2.60E+00**
	std	3.41E+02	5.17E+02	3.68E+02	4.54E+02	**2.97E+02**	3.79E+02	5.38E+02	5.50E+02	7.38E+02	6.26E+02	4.46E+02
	f-rank	11	7	3	2	9	8	4	10	6	5	1
$F_{12}$	mean	**1.10E-02**	**1.10E-02**	5.44E-01	3.55E-01	1.01E+01	1.01E+01	1.01E+01	1.01E+01	3.77E-01	6.33E-01	3.24E-01
	std	**1.74E-18**	**1.74E-18**	1.65E-01	5.72E-02	3.38E-01	3.51E-01	5.26E-01	1.09E+00	1.71E-01	3.36E-01	1.57E-01
	f-rank	1	1	6	4	8	9	10	11	5	7	3
$F_{13}$	mean	6.55E-01	6.78E-01	2.80E-01	3.77E-01	0	0	**0**	0	5.13E-01	5.51E-01	2.63E-01
	std	1.56E-01	7.98E-02	6.30E-02	7.10E-02	2.55E-01	3.63E-01	6.24E-02	1.75E-01	1.41E-01	8.94E-02	**5.61E-02**
	f-rank	10	11	6	7	3	4	1	2	8	9	5
$F_{14}$	mean	6.20E-01	3.93E-01	4.23E-01	2.71E-01	1.00E+02	6.00E+01	**0**	1.00E+01	4.41E-01	2.34E+00	1.83E-01
	std	2.96E-01	1.55E-01	2.15E-01	5.12E-02	1.15E+01	1.22E+01	9.85E-02	1.88E+00	2.46E-01	3.31E+00	**2.11E-02**
	f-rank	7	4	5	3	11	10	1	9	6	8	2
$F_{15}$	mean	1.55E+01	1.88E+01	8.81E+00	1.06E+01	6.84E+05	2.39E+03	**0**	8.00E+01	3.78E+01	8.72E+01	5.97E+00
	std	5.49E+00	5.64E+00	1.51E+00	3.71E+00	2.85E+05	1.22E+03	**1.34E+00**	3.02E+01	9.26E+01	1.01E+02	1.94E+00
	f-rank	5	6	3	4	11	10	1	8	7	9	2
$F_{16}$	mean	1.07E+01	1.06E+01	1.04E+01	9.21E+01	2.02E+01	2.02E+01	2.02E+01	2.00E+01	1.25E+01	1.16E+01	**1.04E+01**
	std	5.83E-01	6.25E-01	6.10E-01	7.30E-01	2.12E-01	3.66E-01	7.64E-01	**1.74E-01**	5.91E-01	6.91E-01	5.14E-01
	f-rank	4	3	2	11	8	9	10	7	6	5	1
$F_{17}$	mean	1.49E+06	1.27E+06	5.71E+05	1.75E+05	9.74E+06	2.53E+06	7.69E+04	1.15E+07	2.80E+04	9.56E+05	**5.63E+00**
	std	9.34E+05	5.46E+05	4.10E+05	1.62E+05	2.79E+06	3.34E+06	8.38E+04	4.59E+06	2.24E+04	7.62E+05	**3.15E+00**
	f-rank	8	7	5	4	10	9	3	11	2	6	1
$F_{18}$	mean	2.89E+03	8.22E+02	6.51E+03	3.89E+03	6.16E+08	1.66E+08	3.30E+03	1.10E+08	3.54E+04	1.47E+05	**5.23E+02**
	std	4.27E+03	1.01E+03	4.62E+02	5.15E+03	1.68E+08	1.03E+08	3.52E+03	4.66E+07	1.62E+05	9.00E+05	**2.11E+02**
	f-rank	3	2	6	5	11	10	4	9	7	8	1
$F_{19}$	mean	5.17E+03	7.80E+03	1.14E+01	7.79E+01	1.90E+02	1.20E+02	1.05E+01	4.00E+01	1.11E+01	2.27E+01	**6.26E+00**
	std	5.67E+03	4.65E+03	2.03E+00	1.78E+00	3.42E+01	3.82E+01	1.74E+00	5.90E+00	1.08E+01	1.43E+01	**6.38E-01**
	f-rank	10	11	4	7	9	8	2	6	3	5	1
$F_{20}$	mean	2.61E+04	1.62E+04	6.27E+02	4.98E+03	3.58E+04	1.68E+04	2.10E+02	1.03E+06	3.02E+03	4.23E+03	**1.49E+02**
	std	1.57E+04	4.11E+03	1.12E+03	3.40E+03	1.76E+04	6.57E+03	8.17E+01	9.05E+05	7.25E+03	3.81E+03	**3.04E+01**
	f-rank	9	7	3	6	10	8	2	11	4	5	1
$F_{21}$	mean	1.11E+06	1.22E+06	2.57E+05	8.91E+04	2.52E+06	2.30E+06	2.70E+04	5.66E+06	5.08E+04	2.33E+05	**1.05E+00**
	std	7.95E+05	7.96E+05	1.76E+05	1.06E+05	1.17E+06	1.35E+06	1.83E+04	2.73E+06	1.22E+05	2.38E+05	**5.92E+00**
	f-rank	7	8	6	4	10	9	2	11	3	5	1
$F_{22}$	mean	1.88E+03	1.66E+02	2.07E+02	2.75E+02	1.02E+03	8.40E+02	2.10E+02	1.34E+03	6.81E+02	3.39E+02	**7.20E+01**
	std	2.03E+02	2.45E+02	2.09E+02	1.45E+02	1.88E+02	1.28E+02	1.01E+02	1.88E+02	2.11E+02	1.78E+02	**4.91E+01**
	f-rank	3	2	4	6	10	9	5	11	8	7	1
$F_{23}$	mean	4.11E+02	3.42E+02	3.14E+02	3.14E+02	6.00E+02	3.90E+02	3.20E+02	3.50E+02	3.14E+02	3.28E+02	**2.00E+02**
	std	6.43E+01	2.84E+01	2.76E-01	1.60E+01	6.70E+01	2.47E+01	4.78E-01	7.66E+00	1.20E-01	4.02E+00	**0**
	f-rank	10	7	3	4	11	9	5	8	2	6	1
$F_{24}$	mean	1.47E+04	3.41E+04	2.02E+02	2.01E+02	4.01E+02	2.30E+02	2.40E+02	2.20E+02	2.46E+02	2.01E+02	2.00E+02
	std	8.37E+03	2.35E+04	3.03E-03	**1.50E-03**	1.42E+01	2.53E+01	6.46E+00	2.51E+00	5.69E+00	1.55E-03	1.60E-03
	f-rank	10	11	4	1	9	6	7	5	8	2	3
$F_{25}$	mean	5.29E+02	6.53E+02	2.03E+02	2.00E+02	2.40E+02	2.10E+02	2.11E+02	2.20E+02	2.08E+02	2.11E+02	2.00E+02
	std	4.36E+01	6.01E+01	1.17E+00	8.07E-01	6.00E+00	1.06E+01	2.08E+00	4.44E+00	4.51E+00	2.82E+00	**0**
	f-rank	10	11	3	2	9	5	6	8	4	7	1
$F_{26}$	mean	2.12E+00	3.64E+01	1.01E+02	1.01E+02	1.01E+02	1.01E+02	1.00E+02	1.01E+02	1.02E+02	1.02E+02	1.27E+02
	std	3.46E+00	5.62E+01	7.36E-02	9.55E-02	2.09E-01	4.30E-01	**6.00E-02**	2.06E-01	1.17E+00	1.53E-01	4.48E+01
	f-rank	10	11	2	3	5	6	1	4	8	7	9
$F_{27}$	mean	**1.95E+02**	3.02E+02	4.08E+02	5.43E+02	1.08E+03	1.29E+03	5.80E+02	5.30E+02	8.82E+02	8.19E+02	2.00E+02
	std	1.04E+02	1.60E+02	6.07E+00	1.35E+02	2.80E+02	3.30E+01	1.41E+02	7.92E+01	2.03E+02	9.17E+01	**0**
	f-rank	2	3	4	6	10	11	7	5	9	8	1
$F_{28}$	mean	1.94E+03	2.12E+03	4.34E+02	9.68E+02	1.39E+03	1.67E+03	9.71E+02	1.44E+03	1.29E+03	9.68E+02	**2.00E+02**
	std	1.04E+02	4.44E+02	8.45E+00	4.12E+01	1.33E+02	2.32E+02	2.43E+02	9.46E+02	2.88E+02	1.06E+02	**0**
	f-rank	10	11	2	3	7	9	5	8	6	4	1
$F_{29}$	mean	1.98E+07	3.09E+07	2.14E+02	5.70E+05	5.70E+06	7.47E+06	1.21E+03	1.20E+06	4.81E+06	1.19E+06	**2.00E+02**
	std	3.95E+06	6.91E+06	2.37E+00	2.14E+06	3.33E+06	1.20E+06	2.16E+02	7.03E+05	4.82E+06	3.25E+06	**0**
	f-rank	10	11	2	4	8	9	3	6	7	5	1
$F_{30}$	mean	6.95E+06	1.38E+07	6.69E+02	2.38E+05	1.49E+05	1.89E+05	4.08E+03	7.66E+04	1.74E+04	1.93E+04	**2.00E+02**
	std	1.03E+07	1.08E+07	2.14E+02	1.10E+03	5.55E+04	1.03E+05	1.44E+03	3.36E+04	3.17E+04	8.25E+03	**4.85E+00**
	f-rank	10	11	2	9	7	8	3	6	4	5	1
Average f-rank		6.80	6.40	3.80	4.30	9.37	8.60	3.97	8.17	5.67	6.50	2.37
Overall f-rank		8	6	2	4	11	10	3	9	5	7	1

### Real world problem: Frame structure design

The design of frame structures is one of the most significant challenges in structural engineering and offers a wide range of design flexibility^141,142^.

The general equation for optimal frame design is expressed as

19 $$\begin{aligned} Find \hspace{10pt} S= [s_1, s_2,...,s_{dv}] \end{aligned}$$

20 $$\begin{aligned} F to \hspace{5pt} minimize \hspace{5pt} f(S)=g(S)\times g_{penalty}(S) \end{aligned}$$

For *W* sections, *S* is the design vector of cross-sectional areas; *f*(*S*) represents the merit functions; *dv* is the number of design variables; *g*(*S*) is the objective function defined as the volume or weight of the frame structure; and $g_{penalty}(S)$is a penalty function resulting from constraint violations on the structural response^142^.

The weight of the frame structure, represented as a function *g*(*S*), is given by

21 $$\begin{aligned} g(S)=\sum _{z=1}^{nm} \gamma _{z} . S_{z} . L_{z} \end{aligned}$$

where *nm* denotes the total number of members comprising the frame; $L_{z}$ represents the length of the *z*-th member in the frame; and $\gamma _z$ is the density of the material in the *z*-th member.

The penalty function, $g_{penalty}(S)$, is defined as follows^143^:

22 $$\begin{aligned} g_{penalty}(S)=(1+ \epsilon _1 . v)^{\epsilon _2}, \hspace{10pt} v=\sum _{z=1}^n max [0, y_z] \end{aligned}$$

where *n* represents the number of constraints in the design problem, $\epsilon _1$ and $\epsilon _2$ are constants derived from *expl* and *expt*, and $y_z$ denotes the displacement or stress constraint. If $y_z$ is positive, its value is added to the constraint functions. These constraints comprise of

Element stresses

23 $$\begin{aligned} y_z^{\sigma }=1-\Big |\frac{\sigma _z}{\sigma _z^a}\Big |\le 0, \hspace{10pt} z=1,2,...,nm \end{aligned}$$

Maximum latent displacement

24 $$\begin{aligned} v^{\Delta }=R-\frac{\Delta T}{H} \le 0 \end{aligned}$$

Inter-story displacements

25 $$\begin{aligned} v_j^d=R_I-\frac{d_j}{h_j}\le 0, \hspace{10pt} j=1,2,...,ns \end{aligned}$$

where $\sigma _z$ represents the stress in the $z^{th}$ member, and $\sigma _z^a$ is the allowable stress in the same member. $\Delta T$ denotes the maximum latent displacement. The total number of stories is denoted by *ns*. The maximum drift index is represented by *R*, and $d_j$ signifies the inter-story drift at the $j^{th}$ floor. The height of the frame structure is *H*, and $h_j$ is the height of the $j^{th}$ story. The inter-story drift index permitted by AISC 2001, indicated by $R_I$, is set to 1/300 as per^143^. The constraints per the LRFD interaction formulas from AISC 2001 are given by:

26 $$\begin{aligned} y_z^Z= {\left\{ \begin{array}{ll} 1-\frac{P_u}{2\phi _c P_n}-\left( \frac{M_{ux}}{\phi _bM_{nx}+\frac{M_{uy}}{\phi _bM_{ny}}}\right) \le 0; \hspace{1pt} For \frac{P_u}{\phi _cP_n} < 0.2 \\ 1-\frac{P_u}{\phi _c P_n}-\frac{8}{9}\left( \frac{M_{ux}}{\phi _bM_{nx}+\frac{M_{uy}}{\phi _bM_{ny}}}\right) \le 0; \hspace{1pt} For \frac{P_u}{\phi _cP_n} \ge 0.2 \end{array}\right. } \end{aligned}$$

where $P_u$ represents the required axial strength, while $P_n$ denotes the nominal axial strength in either tension or compression. The resistance factors are $\phi _t = 0.9$ for tension and $\phi _c = 0.85$ for compression. The flexural resistance reduction factor is $\phi _b = 0.90$. The terms $M_{ux}$ and $M_{uy}$ indicate the required flexural strengths in the *x* and *y* directions, respectively, while $M_{nx}$ and $M_{ny}$ represent the nominal flexural strengths in these directions. For a two-dimensional structure, the value of $M_{ny}$ is set to 0.

The effective length factor *K* is essential for determining Euler and compression stresses. For bracing and beam members, *K* is set to 1. For column members, *K*is calculated using SAP2000. In a generalized case, the approximate effective length, accurate within −1.0% to +2.0%, is based on Dumonteil^144^ and is given by:

27 $$\begin{aligned} K= {\left\{ \begin{array}{ll} \sqrt{\frac{1.6G_AG_B+4(G_A+G_B)+7.5}{G_A+G_B+7.5}}; \hspace{5pt} For \hspace{2pt} unbraced \hspace{2pt} members \\ \frac{3G_AG_B+1.4(G_A+G_B)+0.64}{3G_AG_B+2(G_A+G_B)+1.28}; \hspace{10pt} For \hspace{2pt} braced \hspace{2pt} members \end{array}\right. } \end{aligned}$$

where $G_A$ and $G_B$ are the stiffness ratios of the columns to girders at the two end joints *A* and *B* of the column section, respectively.

In this subsection, MaCN’s performance has been evaluated for the complex real problem of steel frame structure^143^^145^. The weight minimization in this problem is the main objective of the optimization algorithm. The optimized results of MaCN are compared with other algorithms for three frame structures such as $1-bay$ $8-story$, $3-bay$ $15-story$ and $3-bay$ $24-story$. To obtain statistical results for these frame design problems, the algorithm is simulated for 20 independent runs. Although these frame structure problems are rarely used, due to various challenges that have been observed in describing the problem’s clear objective function according to design codes. Here, the main aim of doing comparison among the optimization algorithms is to check the algorithm’s robustness in terms of accuracy, and stopping criteria are maximum iterations. The description of three steel frame problems and the analysis of the results are given in consecutive subsections.

#### 1-bay 8-story

**Fig. 8:**
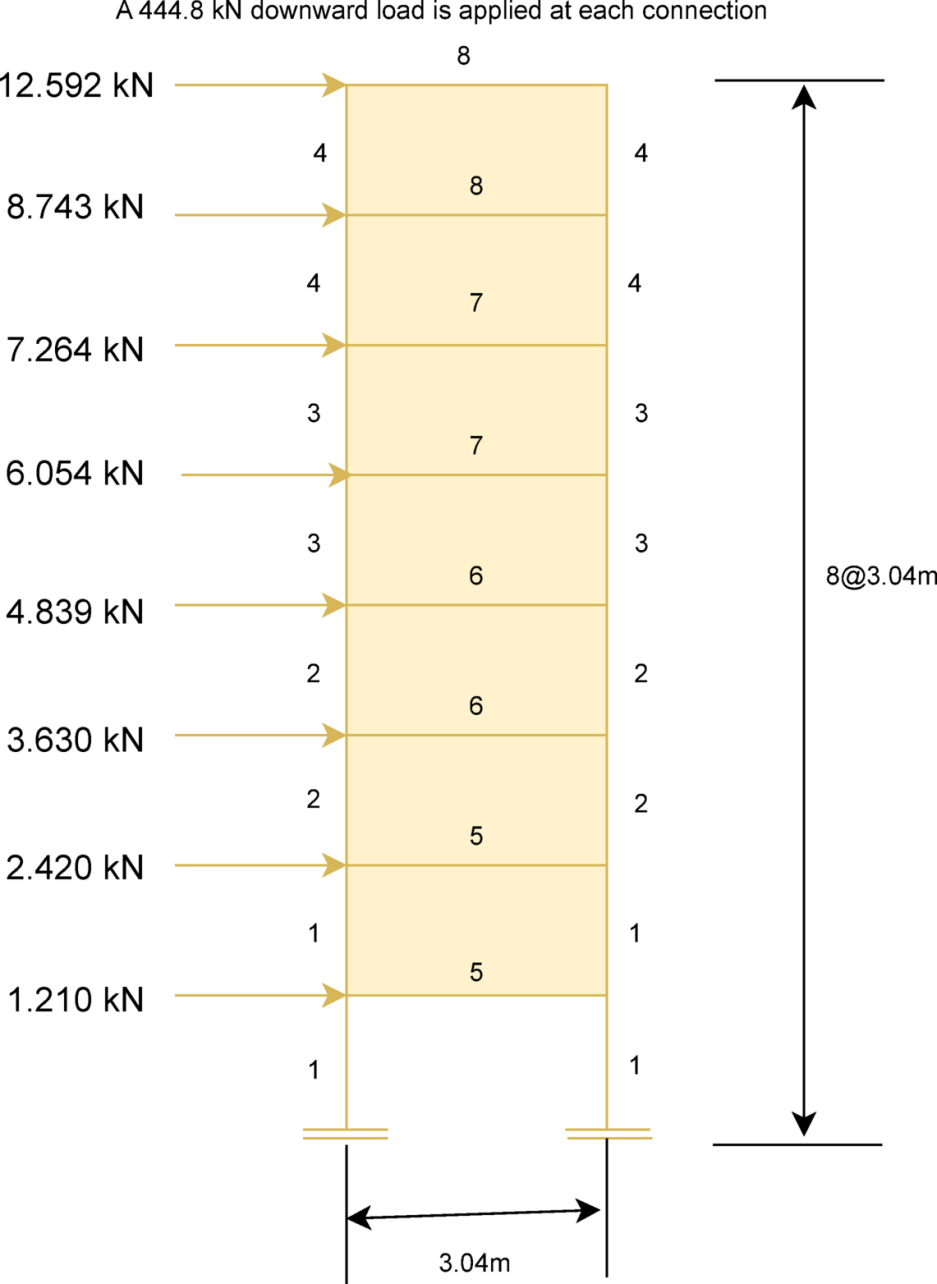
Structure of 1-bay 8-story frame with loading (adapted from^143^).

The fabrication of $1-bay$ $8-story$ frame structure follows the condition of using a single beam during two successive stories, starting from the bottom of the frame. After that, two successive stories have been categorized into a single section of the column. Figure 8 describes the structure of this problem along with various loads that have been implemented on this structure. This frame design problem is considered a constrained optimization problem with one constraint variable, such as lateral drift at the peak point. The value of this lateral drift has to be lower than 5.08 cm, and the elastic modulus (*E*) of the structure is set to 200 GPa. Further, all the structural elements are combined into various categories for the generation of 4 sections of columns and 4 sections of the beam. The cross section area for all these elements must be selected from 267 $W-shaped$ sections.

*Inferences from results:* Table 8 provides the optimized results of MaCN for $1-bay$ $8-story$frame structure problem and comparison has been performed with respect to other algorithms namely DE^143^, ES-DE^143^, genetic algorithm (GA)^146^, ant colony optimization (ACO)^147^, PSOPC^148^, PSOACO^148^, hybrid GA with particle swarm optimization (HGAPSO)^149^and SFLAIWO^150^. From the results, it has been analyzed that proposed MaCN is capable to generate the optimum weight of 30.70 kN and considered as best design algorithm for this problem. Here, it should be noted that optimal weight of MaCN (30.70 kN) is lighter than 1.14 % of optimal weight (31.05 kN) generated by second best algorithm ACO. So, overall it can be say that proposed MaCN is more reliable among all the other algorithms under comparison.

**Table 8:** Optimization results for the 1-bay 8-story frame.

Groups	Optimal W-shaped sections								
	SFLAIWO	DE	PSOACO	PSOPC	ES-DE	ACO	GA	HGAPSO	MaCN
	^150^	^143^	^148^	^148^	^143^	^147^	^146^	^149^	
1	W18X40	W16X36	W18X35	W18X35	W18X40	W21X44	W18X35	W18X35	W21X44
2	W18X35	W16X36	W16X32	W14X26	W18X35	W18X35	W18X35	W18X35	W16X31
3	W14X22	W14X22	W14X22	W16X26	W14X22	W18X35	W18X35	W14X22	W14X22
4	W12X14	W12X22	W12X16	W14X16	W12X14	W12X22	W18X26	W12X16	W12X14
5	W18X35	W18X35	W21X48	W24X62	W18X46	W18X40	W18X46	W16X31	W14X22
6	W18X35	W16X31	W18X40	W18X35	W18X35	W16X26	W16X31	W21X44	W18X40
7	W18X35	W18X40	W16X31	W16X31	W18X35	W16X26	W16X26	W18X35	W18X40
8	W14X22	W14X30	W16X36	W12X30	W12X19	W12X14	W12X16	W16X26	W14X26
Weight (kN)	31.08	32.76	32.29	34.21	31.77	31.05	32.83	31.24	30.70

#### 3-bay 15-story

**Fig. 9:**
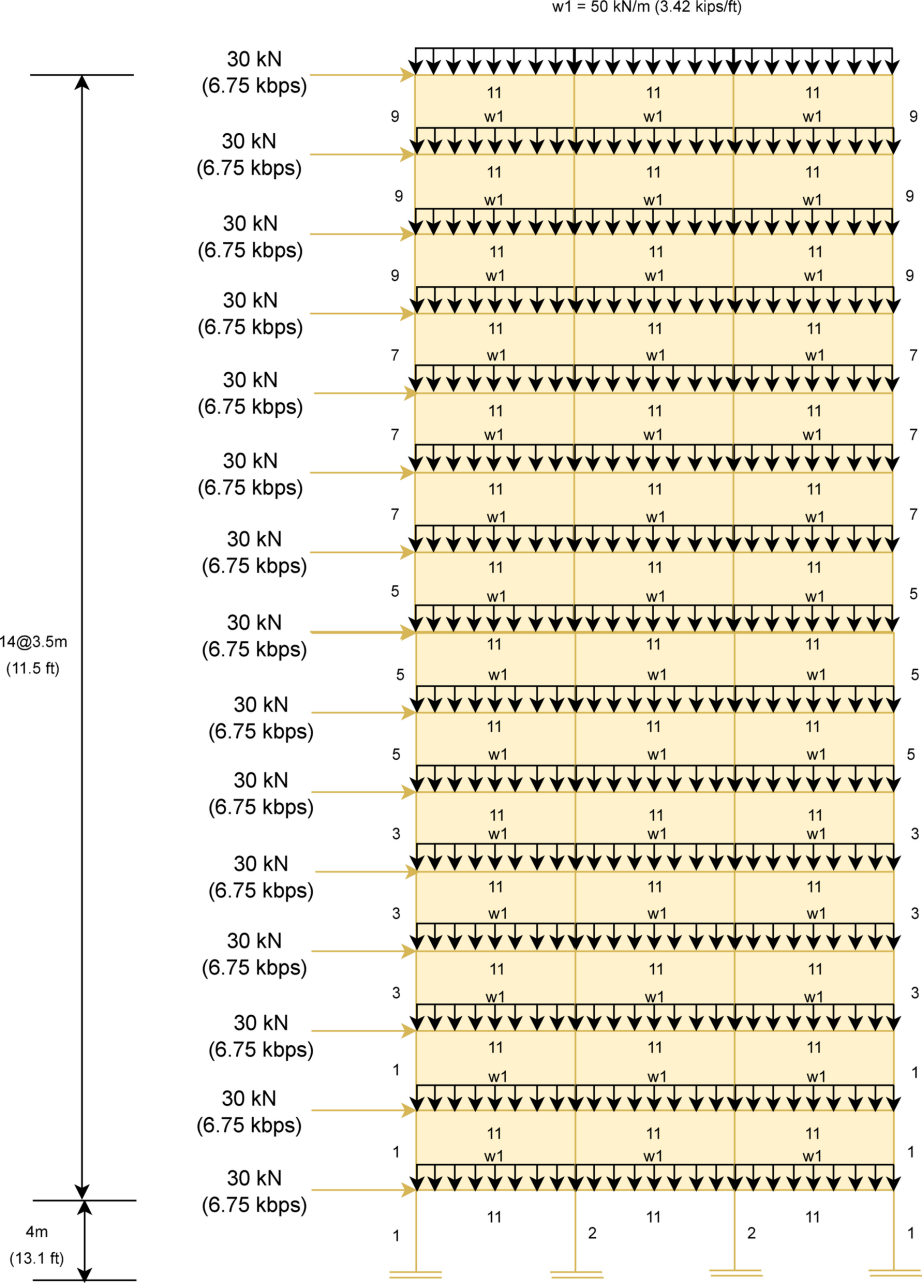
Structure of 3-bay 15-story frame with loading (adapted from^143^).

The structure of $3-bay$ $15-story$ steel frame is schematically shown in Figure 9. This figure also describes the various groups of elements and incorporates various loads to the frame structure. This frame design problem mainly comes with two constraints of optimization, the first constraint is related to displacement and the second constraint deals with the strength of the material provided by the American Institute of Steel Construction (AISC). In the present work, the value of frame top story’s sway should be kept lower than 23.5 cm, the value of material modulus of elasticity (*E*) is equal to 200 GPa and the value of yield stress $(S_y)$is kept constant at 248.2 MPa which are same as^143^. After that, the effective length of frame members for the sway-permitted frame is determined as $L_x$ $\ge$ 0 and for out of a plane is defined as $L_y$ $=$ 1. Here, it should be taken care that all groups of elements are decided from 267 *W* sections, and every column of the frame is taken as non-braced with its length. This non-braced length of every beam is calculated as $1/5^{th}$ length of the span.

*Inferences from results:* The optimized results obtained by various algorithms for $3-bay$ $15-story$ steel frame problem is presented in Table 9. Here twelve improved algorithms are used for comparison with respect to the proposed MaCN algorithm. These algorithms are hybrid harmony search, particle swarm and ant colony optimization (HPSACO)^151^, hybridization of big bang-big crunch algorithm (HBB-BC)^152^, imperialist competitive algorithm (ICA)^153^, DE^143^, ES-DE^143^, accelerated version of water evaporation optimization (AWEO)^141^, enhanced vibrating particle system algorithm (EVPS)^141^, simplified dolphin echolocation (SDE)^141^, SFLAIWO^150^, morlet wavelet mutation based colliding bodies optimization (MWQI-CBO)^154^, set-theoretical multi-phase shuffle shepherd optimization algorithm (STMP-SSOA)^155^and tribe-interior search algorithm (Tribe-ISA)^156^. From the results presented in Table 9, MaCN is found to be best algorithm for structure design of steel frame with optimal weight of 361.14kN. The optimal weight generated by MaCN is lighter than $5\%$ from the weight (379.21kN) generated by second most promising algorithm SFLAIWO. Hence, MaCN is again capable to provide superior performance for this problem because heavier optimal weights are given by all other algorithms under test.

**Table 9:** Optimization results for the 3-bay 15-story frame.

Groups	Optimal W-shaped sections												
	HPSACO	ICA	HBB-BC	DE	AWEO	ES-DE	EVPS	SDE	SFLAIWO	MWQI-CBO	STMP-SSOA	Tribe-ISA	MaCN
	^151^	^153^	^152^	^143^	^141^	^143^	^141^	^141^	^150^	^154^	^155^	^156^	
1	W21X111	W24X117	W24X117	W21X122	W18X143	W18X106	W14X99	W14X90	W14X90	W14X90	W16X89	W14X109	W21X55
2	W18X158	W21X147	W21X132	W33X141	W24X162	W36X150	W27X161	W36X170	W26X146	W36X170	W36X170	W24X146	W30X90
3	W10X88	W27X84	W12X95	W14X82	W24X84	W12X79	W24X84	W27X84	W18X76	W27X84	W12X79	W18X86	W21X57
4	W30X116	W27X114	W18X119	W30X108	W33X118	W27X114	W24X104	W24X104	W24X104	W24X104	W27X114	W18X97	W8X67
5	W21X83	W14X74	W21X93	W30X108	W12X65	W30X90	W14X61	W14X61	W12X72	W14X61	W24X68	W14X74	W12X45
6	W24X103	W18X86	W18X97	W12X79	W18X97	W10X88	W30X90	W30X90	W18X86	W30X90	W18X86	W18X86	W14X61
7	W21X55	W12X96	W18X76	W14X61	W12X50	W18X71	W14X48	W14X48	W12X58	W14X48	W14X48	W24X68	W16X36
8	W27X114	W24X68	W18X65	W18X71	W21X68	W18X65	W12X65	W12X65	W14X61	W14X61	W14X61	W21X55	W14X34
9	W10X33	W10X39	W18X60	W6X25	W8X28	W8X28	W6X25	W6X25	W6X25	W14X34	W12X30	W8X24	W5X19
10	W18X46	W12X40	W10X39	W24X62	W16X40	W12X40	W12X40	W12X40	W16X36	W8X35	W10X39	W10X33	W8X28
11	W21X44	W21X44	W21X48	W21X48	W21X44	W21X48	W21X44	21X44	W21X44	W21X44	W21X44	W21X48	W18X28
Weight(kN)	426.36	417.47	434.54	423.83	429.46	415.06	389.77	387.89	379.21	386.63	389.08	399.27	**361.14**

#### 3-bay 24-story

**Fig. 10:**
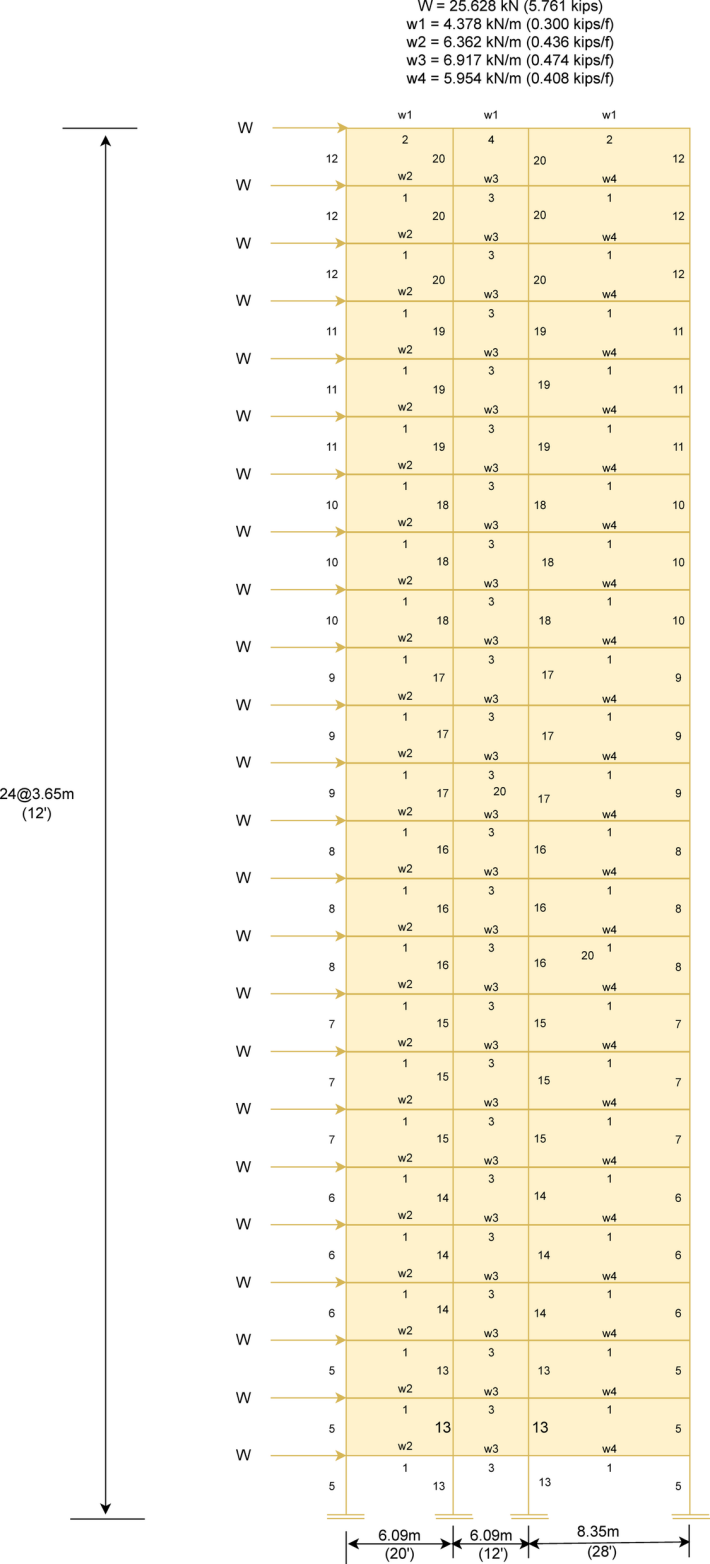
Structure of 3-bay 24-story frame with loading (adapted from^143^).

The design of $3-bay$ $24-story$steel frame was firstly described by^157^ and comprised a total of 168 members. These members are made up by using a total number of 96 columns and 72 beams. Figure 10 shows the schematic of this steel frame along with topology and various types of loads utilized on this frame structure. Here, it should be noted that the steel frame design must follow all the specifications provided by LRFD of AISC. This frame design has a displacement constraint treated as an optimization constraint under an inter-story drift with an index less than 1/300. The value of elastic modulus (*E*) and yield of stress $(S_y)$ is set to 205 GPa and 230.3 MPa, respectively.

Further, the effective length of frame members is calculated with factor $L_x \ge 0$ in the sway-permitted frame and $L_y =1$ for out of the plane. In order to fabricate the frame, some conditions have to be followed in which $1^{st}$ and $3^{rd}$ bay on all floors other than the roof’s beam must be handled by a single section of the beam and create 4, beam groups. Apart from that, one group is associated with outer columns, and another group is associated with inner columns of the frame over 3 following stories. So, it can be said that this problem has 16 column groups and 4 beam groups for the total number of 20 design variables.

*Inferences from results:* For $3-bay$ $24-story$frame design problem, optimized results of MaCN along with other algorithms namely ACO^158^, DE^143^, HBB-BC^152^, harmony search (HS)^144^, improved ACO (IACO)^147^, ICA^153^, hybrid bang-bing crunch PSO (HBBPSO)^159^, ES-DE^143^, AWEO^141^, EVPS^141^, SFLAIWO^150^, improved electro-search (IES) algorithm^160^and Tribe-ISA^156^ are provided in Table 10. Here, EVPS provided the best optimal weight of 905.67kN which is marginal 0.3% lighter than proposed MaCN optimal weight 908.41kN. In the present scenario, it has been found that structural weights reported in literature by other algorithms are heavier than MaCN except EVPS. So, overall EVPS and MaCN are found to be capable for providing a solution near to global optimal solution.

**Table 10:** Optimization results for the 3-bay 24-story frame.

Groups	Optimal W-shaped sections													
	ACO	DE	Tribe-ISA	HS	IES	HBB-BC	ICA	IACO	HBBPSO	ES-DE	AWEO	EVPS	SFLAIWO	MaCN
	^158^	^143^	^160^	^144^	^150^	^152^	^153^	^147^	^159^	^143^	^141^	^141^	^156^	
1	W30X90	W30X90	W27X84	W30X90	W30X90	W30X90	W30X99	W30X90	W30X90	W30X90	W30X90	W30X90	W30X90	W30X90
2	W8X18	W21X48	W21X50	W10X22	W6X15	W21X18	W21X50	W16X26	W21X55	W21X55	W8X18	W6X15	W21X48	W18X60
3	W24X55	W21X44	W24X68	W18X40	W24X55	W18X46	W24X55	W18X35	W21X48	W21X48	W24X55	W24X55	W21X48	W24X55
4	W8X21	W27X129	W8X21	W12X16	W6X9	W8X21	W8X28	W14X22	W27X24	W10X45	W26X8.5	W6X8.5	W21X48	W16X36
5	W14X145	W14X176	W14X109	W14X176	W14X159	W14X176	W14X109	W14X145	W14X176	W14X145	W14X193	W14X159	W12X19	W14X159
6	W14X132	W14X120	W14X109	W14X176	W14X132	W14X159	W14X159	W14X132	W14X90	W14X109	W14X120	W14X145	W14X176	W14X132
7	W14X132	W14X132	W14X109	W14X132	W14X109	W14X109	W14X120	W14X120	W14X99	W14X99	W14X132	W14X90	W14X109	W14X109
8	W14X132	W14X132	W14X74	W14X109	W14X99	W14X90	W14X90	W14X109	W14X99	W14X145	W14X82	W14X74	W14X109	W14X90
9	W14X68	W14X109	W14X90	W14X82	W14X68	W14X82	W14X74	W14X48	W14X74	W14X109	W14X61	W14X74	W14X90	W14X68
10	W14X53	W14X53	W14X53	W14X74	W14X61	W14X74	W14X68	W14X48	W14X74	W14X48	W14X38	W14X38	W14X48	W14X61
11	W14X43	W14X61	W14X34	W14X34	W14X30	W14X38	W14X30	W14X34	W14X38	W14X38	W14X34	W14X30	W14X30	W14X30
12	W14X43	W14X30	W14X30	W14X22	W14X22	W14X30	W14X38	W14X30	W14X34	W14X30	W14X22	W14X22	W14X34	W14X30
13	W14X145	W14X99	W14X132	W14X145	W14X90	W14X159	W14X159	W14X159	W14X145	W14X99	W14X82	W14X99	W14X90	W14X90
14	W14X145	W14X132	W14X132	W14X132	W14X99	W14X132	W14X132	W14X120	W14X132	W14X132	W14X109	W14X90	W14X120	W14X99
15	W14X120	W14X109	W14X120	W14X109	W14X90	W14X109	W14X99	W14X109	W14X109	W14X109	W14X82	W14X99	W14X99	W14X90
16	W14X90	W14X74	W14X120	W14X82	W14X74	W14X82	W14X82	W14X99	W14X90	W14X68	W14X82	W14X90	W14X90	W14X82
17	W14X90	W14X82	W14X74	W14X61	W14X68	W14X68	W14X68	W14X82	W14X74	W14X68	W14X68	W14X68	W14X61	W14X68
18	W14X61	W14X82	W14X61	W14X48	W14X43	W14X48	W14X48	W14X53	W14X48	W14X68	W14X68	W14X61	W14X53	W14X43
19	W14X30	W14X48	W14X38	W14X30	W14X34	W14X34	W14X34	W14X38	W14X38	W14X61	W14X43	W14X43	W14X34	W14X43
20	W14X26	W14X82	W14X22	W14X22	W14X22	W14X26	W14X22	W14X26	W14X22	W14X22	W14X34	W14X22	W14X22	W14X22
Weight(kN)	980.63	997.56	927.15	956.13	929.10	960.90	946.25	967.33	941.55	945.15	927.59	909.67	911.78	**908.41**

## Discussion of results

This section provides an extensive summary of the results, along with some advantages of the proposed MaCN algorithm over others. It also discusses the specific drawbacks and some future insights on how we can mitigate them in the future.

### Summary of results

In summary, the proposed MaCN resulted in a set of excellent and stimulating results in this field to solve complex mathematical and engineering design problems. These results reinforce the goal of developing a new way to find solutions that are better than the available solutions through an advanced method of searching for optimal solutions.

In addition, the strategies used can offer a more satisfying equilibrium between the search strategies (*expl* and *expt*), especially in the last phase. Such techniques help the cases in which the search space is constrained, and unnecessary *expl* can reduce the convergence speed-up of an algorithm without changing the quality of solutions. Moreover, the proposed MaCN got all the core advantages of CS, NMRA, bare-bones search operators, and parametric adaptations together.

This is further complemented by the use of numerical benchmarks and engineering frame design problems. From the experimental results, it has been found that for CEC 2005, MaCN has superior performance over JADE, SaDE, CMA-ES, SHADE, LSHADE-SPACMA, MGSCA, FA-FPO, GWO-E and OEWOA. All of these comparative algorithms are hybrid versions of well-known algorithms and have been proposed recently. In CEC 2014 problems, performs better compared to LX-BBO, ISOS, VNBA, MGSCA, RW-GWO, B-BBO, IMEHO, CCS, BDE and other; again proving the superiority of the proposed algorithm. In addition to these comparisons, the statistical results for $1-bay$ $8-story$, $3-bay$ $15-story$, and $3-bay$ $24-story$ show that MaCN is better than SFLAIWO, PSOACO, PSOPC, ES-DE, HGAPSO, EVPS, MWQL-CBO, STMP-SSOA, Tribe-ISA, HBB-BC, AWEO, ICA and others. It is included in better solutions and more accelerated convergence. These results are complemented by statistical tests, which further demonstrate the superiority of the proposed MaCN in finding better candidate solutions relative to others.

### Limitations of the proposed approach

In view of all practical advantages, there are also some limitations. The integrated method using three strategies effectively enhances the quality of candidate solutions. Increases the performance of conventional CS, while requiring more time to achieve the optimal solution. Therefore, there must be a careful trade-off between accuracy and performance when addressing real problems.

Although the population reduces over subsequent iterations and this helps in reducing the overall complexity of the algorithm, an initial and a final population needs to be defined to start the exploration process and finally avoid premature convergence due to a very small population in the end. Thus, handling population is still a concern for the proposed approach, and more rigorous studies must be conducted to adapt it.

In addition to that, evaluating fitness functions can be computationally expensive and noisy, further complicating the optimization process. An effective application of MaCN will require problem-specific customizations and significant domain knowledge to design appropriate representations and operators. Furthermore, there is no guarantee of finding the global optimum and its stochastic nature can result in different outcomes across multiple runs.

### Insightful Implications

Despite various limitations, MaCN can be a valuable addition to the optimization toolbox. It can be particularly useful for solving complex, non-linear, and poorly understood problems where traditional methods might fail. By understanding and mitigating the limitations of MaCN through hybrid approaches, dynamic algorithmic tuning, and problem-specific adaptations, its effectiveness can be improved in various application domains. The flexibility of MaCN allows for applications in fields ranging from artificial intelligence and machine learning to engineering design and bioinformatics. It can be used for the optimization of neural network architectures, leading to advancements in deep learning, and facilitate robust design in engineering by efficiently navigating vast and complex design spaces. In addition, MaCN inspires new ways to approach problem solving by emphasizing the importance of diversity, adaptability, and decentralized control, which can lead to more resilient and adaptive systems in various applications.

## Conclusion

A novel version of CS, namely MaCN was proposed to alleviate the drawbacks of basic CS. The proposed MaCN is based on hybridizing the CS with NMRA to overcome local search problems. It is also enhanced with self-adaptive properties to improve *expt* ability. The main idea here is to use iterative division and population division, along with a reducing *popsize* for better search operation with improved properties and less computational complexity. In addition, a bare-bones search operator is used to further enhance *expl* search ability. The adaptive *iw* is also added to optimize the switching probability benefits to obtain the appropriate stability between the search methods (i.e., *expl* and *expt*). The proposed MaCN algorithm is examined to solve the CEC2005 and CEC2014 benchmark test suite and three real-world steel frame design problems (that is, 1-bay 8-story, 3-bay 15-story and 3-bay 24-story). The comparative studies on CEC 2005 and CEC 2014 benchmarks show that MaCN is competitive with respect to JADE, SHADE, LSHADE-SPACMA, SaDE, LX-BBO, IMEHO, among others. In addition to that, experimental and statistical results on industrial engineering frame structure show its superiority compared to PSOPC, SFLAIWO, PSOACO, ES-DE, and others. The results showed that the proposed MaCN obtained encouraging and promising results in solving the CEC competition benchmark test functions compared to other state-of-the-art comment methods. Moreover, it demonstrated its high performance in solving real-world frame design problems by getting excellent results in solving these kinds of complex problem.

For future research, the proposed method can be investigated deeply to see the effect of each component and enhance it by other search operators. We can also incorporate various aspects such as archive based initialization, storing a history of solutions in the algorithm for better search capabilities, and others. An important aspect to look into will be the integration of MaCN with other hybrid approaches and see how these algorithms behave for complex real world problems. Moreover, the proposed methods can be used to solve feature selection, text clustering, image segmentation, task scheduling-based cloud computing; text classification, photovoltaic parameter estimation, constrained optimization problems, text summarization, big data application, image edge detection, networks applications, and other industrial engineering problems.

## Data Availability

The data presented in this study are available on request from the corresponding author.
